# HI-HPTLC-UV/Vis/FLD-HESI-HRMS and bioprofiling of steviol glycosides, steviol, and isosteviol in *Stevia* leaves and foods

**DOI:** 10.1007/s00216-020-02618-4

**Published:** 2020-04-23

**Authors:** Gertrud E. Morlock, Julia Heil

**Affiliations:** Institute of Nutritional Science, Chair of Food Science, and TransMIT Center for Effect-Directed Analysis, Justus Liebig University Giessen, Heinrich-Buff-Ring 26-32, 35392 Giessen, Germany

**Keywords:** Adulteration, Falsification, Food safety, High-performance thin-layer chromatography, Effect-directed analysis, Bioassay

## Abstract

Food products and botanicals on the global market need to be investigated in a more comprehensive way to detect effects, falsifications or adulterations. This is especially true for such ones containing *Stevia* leaves, *Stevia* extracts, or steviol glycosides. A multi-imaging profiling was developed exploiting hydrophilic interaction liquid chromatography (HILIC). A minimalistic sample preparation, different mixtures of acetonitrile and water/buffer on the silica gel phase as well as derivatization reagents and optional hyphenation with high-resolution mass spectrometry were exploited. The hydrophilic interaction high-performance thin-layer chromatography (HI-HPTLC) development took 10 min for 48 analyses. It was used to screen *Stevia* leaf extracts and 20 different food products. For the first time, the biological and biochemical profiling of *Stevia* leaf products by HI-HPTLC-UV/Vis/FLD-assay pointed to 19 different bioactive compound bands found in the more natural multicomponent *Stevia* leaf extracts, whereas most of these activities were not existent for the steviol glycosides. The chemically isolated, purified, and EU-regulated steviol glycosides ease risk assessment and food product development. However, multipotent botanicals may have subtle impact on homeostasis via several metabolic pathways, providing benefits for the consumer’s health. Analyzed side by side by means of the effect-directed profiling, their individual activity profiles were visualized as image and individual substances of importance were pointed out. Multi-imaging (comprehensive detection) plus non-targeted bioprofiling (focus on known and unknown bioactivity) allows for a fast detection of questionable product changes that occur along the global food chain and are particularly related to food safety.

**Electronic supplementary material:**

The online version of this article (10.1007/s00216-020-02618-4) contains supplementary material, which is available to authorized users.

**Figure:**
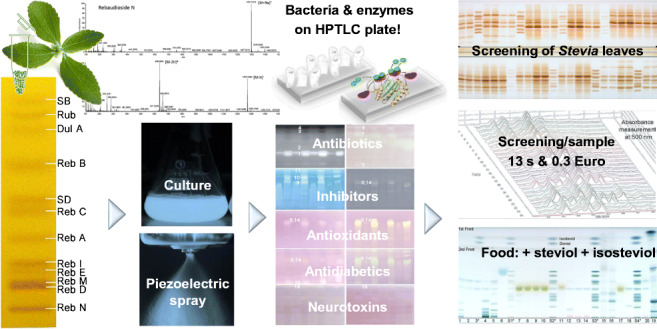
Graphical abstract

## Introduction

The increasing cases of adulteration or falsification of botanicals is challenging food safety worldwide [1–4]. *Stevia* leaves, *Stevia* extracts, and food products or formulations containing the EU-regulated steviol glycosides are also concerned [5], due to their higher price in comparison with synthetic sweeteners. It is known that official methods may lack in detecting adulterations [6], as most analytical methods are tailored with regard to extraction, separation, and detection to the analysis of selected target compounds. The status quo methods by high-performance liquid chromatography tandem mass spectrometry (HPLC-MS/MS or -MRM) confirm such target compounds, but do not necessarily point to other compounds that matter. Often, it is forgotten that not every compound is ionizable by electrospray ionization (ESI) under standard MS settings. This makes, without prior knowledge on potential changes along the global product chain, most analytical mainstream techniques blind for contaminations, residues, adulterations, or other unauthorized additions. Only holistic methods are capable to solve this gap. However, most analytical techniques in this respect are time-consuming, complicated, analyze only a selected part of the sample (due to the required sample preparation), or are not robust for transfer to other laboratories.

Until now, various analytical techniques [7] have been used for the determination of steviol glycosides in food and *Stevia* leaves. Nuclear magnetic resonance (NMR) spectroscopy [8–12] is considered to be powerful for its unbiased overview on most, but not all metabolites present in a sample. Routine NMR lacks in the relevant ability to detect (ultra) trace components, but these do matter when coming along with strong biological or biochemical effects. Furthermore, enzymatic methods [13], electroanalytical techniques [14], capillary electrophoresis [15], desorption electrospray ionization MS [16], paper spray-MS [17], near infrared [18], and Raman spectroscopy [19] were exploited as analytical tools. However, mainly HPLC with different detectors (UV [20], ELSD [21], CAD [22], and MS [23–27]) or high-performance thin-layer chromatography (HPTLC) with different derivatization reagents [5, 28–33] were used. Among these, a fast reversed phase HPLC-MS/MS method was reported for analysis of seven steviol glycosides (not all baseline separated), isosteviol, and steviol in *Stevia* leaf extracts, with a total run time of 9 min per sample [34]. A good resolution of up to 13 steviol glycosides was obtained within 20 min [35]. Samples of a simple composition like table top sweeteners and beverages were directly analyzed after dilution [36–38]. In more complex food, however, the main challenge is the separation of the steviol glycosides from any interfering matrix of similar chemical nature. An additional, tailored sample clean-up was required for each type of food matrix, like dairy products, sauces, or biscuits [39–42]. The workflow strongly depended on the coeluting matrix preset. Depending on the food matrices, the otherwise powerful HILIC separation of steviol glycosides lacks in reproducibility, stable retention times, and constant peak profiles [35]. Matrix-matched calibration, standard addition, or the use of an internal standard were exploited for a proper quantification [43–45]. It is evident that research on analytical techniques is still needed to establish holistic methods for challenging food samples. Also, HPTLC-MS needs to be automated. This has been addressed to manufacturers since 2005 because in a retrospective view, all chromatographic techniques were boosted and did highly benefit upon hyphenation with MS [46]. Fully automated elution head-based and desorption-based devices were built and successfully demonstrated in 2007 [47] and 2018 [48], respectively. Latest innovation was a cost-effective, open-source add-on kit for automating zone elution [49] which was designed to fill the longstanding scientific gap and advance HPTLC-MS.

Presently, steviol glycosides without an off-flavor containing more saccharide moieties, and thus a higher molecular weight, are a hot topic [50]. Produced via microbial fermentation at a higher quantity, these are generally recognized as safe, though not yet in the EU. Steviol and isosteviol are less polar degradation products of steviol glycosides, which may degrade under certain processing and storage conditions. Strongly acidic or fermented heat-treated food matrices may challenge their stability. Such breakdown products should not be present in *Stevia* leaf and food products. Rarely, methods include the analysis of breakdown products [23, 34, 36, 51], although this is of high interest to confirm stability in the different food matrices.

The reliable quantification of steviol glycosides in *Stevia* leaves, *Stevia* extracts, and food products/formulations by planar chromatography was reported recently [5, 33]. Hence, in this study, the analyte focus was extended by such representatives containing a higher number of saccharide moieties. A straightforward method for their screening in different food matrices was intended to be developed, which is able to efficiently analyze products on the global market. *Stevia* plants are differently cultivated and leaf extracts are diversely processed [52]. Therefore, the composition of products can vary and a non-target effect-directed profiling was aimed to be developed for a comprehensive study of potential effects deriving from *Stevia* leave extracts in comparison with steviol glycosides [53, 54]. Such assays with the most important outcome may be selected as assay candidates for future bioprofiling performed in routine with regard to quality control and safety of products.

## Materials and methods

### Chemicals

Bidistilled water was produced by a Heraeus Destamat Bi-18E (Thermo Fisher Scientific, Dreieich, Germany). Purity grades were subsequently listed when they were available. All salts were of p. a. quality and waterfree, if not stated otherwise. 2-Naphthol (≥ 98%) was from Alfa-Aesar, Zürich, Switzerland. Primuline sodium salt (≥ 50%), methanol (MS quality), α-glucosidase from *Saccharomyces cerevisiae* (1000 U/vial), acarbose (for pharm.), tyrosinase from mushroom (≥ 1000 U/mg, 25 kU/vial), β-glucuronidase from *E. coli* (5000 U/vial), acetylcholinesterase from *Electrophorus electricus* (AChE, ≥ 245 U/mg, 10 kU/vial), butyrylcholinesterase from equine serum (≥ 140 U/mg), diammonium hydrogen phosphate (99%), peptone from casein (tryptone, for microbio.), cyclohexane (HPLC grade), sodium acetate, monopotassium phosphate, magnesium sulfate heptahydrate, sodium chloride, Müller-Hinton broth (for microbio.), imidazole (≥ 99.5%), and 2,2-diphenyl-1-picrylhydrazyl (DPPH^•^, 95%) were delivered by Fluka, Sigma-Aldrich, Steinheim, Germany. 2-Naphthyl-β-d-glucopyranoside (95%) and β-glucosidase from almonds (3040 U/mg) were provided by ABCR, Karlsruhe, Germany. 1-Naphthyl acetate (≥ 98%), and formic acid (≥ 98%) were obtained by AppliChem, Darmstadt, Germany. 2-Naphthyl-α-d-glucopyranoside was delivered by Fluorochem, Hadfield Derbyshire, UK. Fast Blue B salt (95%) was purchased from MP Biomedicals, Eschwege, Germany. 5-Bromo-4-chloro-1*H*-indol-3-yl β-d-glucopyranosiduronic acid (X-Gluc, > 98%) was obtained from Carbosynth, Compton-Berkshire, UK. Ethanol, acetone (all solvents of chromatography grade), bovine serum albumin (BSA, fraction V, ≥ 98%), dipotassium hydrogen phosphate (≥ 99%), sodium dihydrogen phosphate monohydrate (≥ 98%), glycerol (Rotipuran, 86%), potassium dihydrogen phosphate (99%), sodium hydroxide (≥ 98%), disodium hydrogen phosphate (≥ 99%), polyethylene glycol (PEG) 8000 (Ph. Eur.), kojic acid (> 98%), acetic acid (100%), sulfuric acid (96%), hydrochloric acid 37% (HCl, purest), 3-[4,5-dimethylthiazol-2-yl]-2,5-diphenyltetrazolium bromide (MTT, ≥ 98%), gallic acid (≥ 98%), 3-[(3-cholamidopropyl) dimethylammonio]-1-propanesulfonate (CHAPS, ≥ 98%), and tris(hydroxymethyl)aminomethane (Tris, ≥ 99.9%) were from Roth, Karlsruhe, Germany. d-Saccharolactone and (2S)-2-amino-3-(3,4-dihydroxyphenyl) propanoic acid (levodopa) were obtained from Santa Cruz Biotechnology, Dallas, TX, USA. Ethyl acetate (≥ 99.8%) and yeast extract powder (for microbiol.) were purchased from Th. Geyer, Renningen, Germany. The medium for the Gram-negative, naturally luminescent marine *Aliivibrio fischeri* bacteria (DSM-7151, German Collection of Microorganisms and Cell Cultures, Berlin, Germany) is listed elsewhere [55]. Gram-positive soil bacteria *Bacillus subtilis* subsp. spizizenii (DSM-618) as well as HPTLC plates silica gel 60 with and without F_254_ (20 × 10 cm) were provided by Merck, Darmstadt, Germany. Steviolbioside (SB, 88%), rubusoside (Rub, 94%), dulcoside A (Dul A, 89%), rebaudioside (Reb) A (Reb A, 97.3%), Reb B (99.2%), Reb C (91.0%), Reb D (93.7%), Reb N (82%), stevioside (SD, 97.7%), isosteviol (IS, 99%), and steviol (S, 99.0%) were obtained by PhytoLab, Vestenbergsgreuth, Germany. Reb I, Reb E, and Reb M (each 100% purity) were supplied by Wisdom Natural Brands, Gilbert, AZ, USA. Saccharides and further derivatization reagents were reported elsewhere [56–59].

### Stock solutions and standard mixture

Stock solutions of each steviol glycoside, steviol, isosteviol, and saccharide (1 mg/mL each) were prepared in methanol and ultrasonicated for 5 min. A standard mixture was prepared by mixing together 140 μL each of the 12 steviol glycoside stock solutions in a sampler vial (1:12 dilution; 84 ng/μL). All solutions and subsequent extracts were stored at − 20 °C until use.

### Extraction of leaf samples

Four dried, pulverized leaf samples of *Stevia rebaudiana* Bertoni were obtained from H.-W. Koyro, Justus Liebig University, Giessen, Germany, and as listed (see Electronic Supplementary Material (ESM) Table S1). For bioprofiling, each *Stevia* leaf sample was extracted with ethanol-water 4:1 (V/V) as well as ethyl acetate (10 mg/mL each), vortexed (30 s), ultrasonicated (15 min), centrifuged (17,000×*g*, 5 min), and transferred to a sampler vial.

### Extraction of food samples

Twenty food samples purchased on the local market or internet (ESM Table S1) were extracted with methanol-water 9:1, vortexed (30 s), ultrasonicated (15 min), and if needed, centrifuged (17,000×*g*, 5 min) or diluted as listed. Using mortar and pistil, inhomogeneous particulate solid samples (e.g., tea) were grinded before extraction.

### Remarks for the analysis

HPTLC plates were prewashed for HRMS recording or in case of interfering α-/β-fronts. A set of plates was either fully immersed or developed with methanol-water, 4:1 (V/V), up to the upper plate edge in the Simultan Separating Chamber (biostep, Burkhardtsdorf, Germany), and dried in an oven at 110 °C for 20 min [60, 61]. For storage in a desiccator, a clean counter glass plate was placed on top of the stacked plates, which were altogether wrapped in aluminum foil. For the *B. subtilis* bioassay, HPTLC plates without fluorescence indicator were used. As steviol glycosides of high purity are expensive, the application parameters were selected so that no microliter volume was lost for the automated syringe operation (recommended for method development, but not for quantification: fill only programmed volume was check-marked and filling vacuum time was 0 s; Automatic TLC Sampler 4, ATS4, CAMAG, Muttenz, Switzerland). Application on both sides or as track patterns was operated by the FreeMode option of the winCATS software (CAMAG). Plate drying was performed in a stream of cold air (hair dryer or Automatic Developing Chamber 2, ADC 2, CAMAG), immediately after application (0.5 min) and development (3 min). The relative humidity of the ambient air was ≤ 35% during the developments. All instrument operation and the acquired data were processed with the software winCATS (version 1.4.7.2018) or visionCATS (version 2.5.18262.1, both CAMAG).

### HILIC separation (HI-HPTLC)

The solutions were applied on the silica gel layer as follows (ATS4, CAMAG): band length 8 mm (6 mm for screening), track distance 10 mm (7.5 mm for screening), distance from lower edge 8 mm and from left edge at least 14 mm, dosage speed 150 nL/s, application volumes 0.5–10 μL/band (42–840 ng/band each) for standard mixture, 0.5–5 μL/band for steviol and isosteviol (0.5–5 μg/band each), 2–6 μL (1–3 μL for screening) for sample extracts (listed in detail in ESM Table S1). The development was performed with 7 mL acetonitrile-water, 4:1 (V/V), taking 13 min up to a developing distance of 60 mm (measured from the lower plate edge, 20 cm × 10 cm Twin Trough Chamber, biostep, or ADC 2, CAMAG). High-throughput screening was performed with 4 mL mobile phase (for each side, antiparallel) in the horizontal developing chamber, taking 10 min up to 50 mm (20 × 10 cm, Chromdes, Lublin, Poland, or CAMAG).

### 2-Step separation of steviol, isosteviol, and steviol glycosides

Three optional workflows were performed as follows: For workflow I (20 min analysis time, 10 mL solvent consumption), development was first performed with 7 mL acetonitrile-water, 4.5:1 (all V/V), taking 13 min up to 60 mm for separation of steviol glycosides. After plate drying (3 min) and plate cut at 50 mm (plate middle), the upper plate part (steviol and isosteviol in previous solvent front are now positioned at 10 mm) was secondly developed with 3 mL *n*-hexane-glacial acetic acid, 19:1, taking 7 min up 40 mm (plate edge) for separation of steviol and isosteviol.

For workflow II (60 min analysis time, 14 mL solvent consumption), development was first performed with 7 mL *n*-hexane-ethyl acetate-glacial acetic acid 7:3:1, taking 44 min up to 85 mm for separation of steviol and isosteviol, and secondly, with 7 mL acetonitrile-water 4.5:1, taking 13 min up 60 mm for separation of steviol glycosides.

For workflow III (20 min analysis time, 95 mL solvent consumption), start bands were applied at 60 mm (instead of 8 mm) and development was first performed with 88 mL *n*-hexane-ethyl acetate-glacial acetic acid 7:3:1 (20 × 10 cm, Immersion Chamber, Chromacim, Voiron, France, covered by a foil), taking 6 min up to 40 mm (plate edge) for separation of steviol and isosteviol, and secondly after plate cut at 70 mm (plate cutter, biostep or TLC SmartCut, CAMAG) and 180°-turn of the lower plate part (previous application line, still containing the steviol glycosides, now positioned at 10 mm) with 7 mL acetonitrile-water 4.5:1, taking 13 min up to 60 mm.

### Documentation, post-chromatographic derivatization, and densitometry

After development, documentation was performed at UV 254 nm (UV), UV 366 nm (FLD), and under white light illumination (Vis; TLC Visualizer, CAMAG). The derivatization reagents were piezoelectrically sprayed (Derivatizer, CAMAG) using 4 mL each of

1. 2-naphthol sulfuric acid reagent (2 g in 180 mL ethanol plus dropwise 8 mL 50% sulfuric acid, blue nozzle, level 6), followed by plate heating (160 °C, 2 min; TLC Plate Heater, CAMAG) and documentation under white light illumination or
2. primuline reagent (0.1 g in 40 mL water plus 160 mL acetone, yellow nozzle, level 6), followed by plate drying (2 min) and documentation at UV 366 nm or
3. anisaldehyde sulfuric acid reagent (1 mL 4-methoxybenzaldehyd, 8 mL sulfuric acid, 16 mL glacial acetic acid and 140 mL methanol, blue nozzle, level 3), followed by plate heating (110 °C, 5 min; or 160 °C, 2 min) and documentation under white light illumination.

The densitometric measurement (TLC Scanner 4, CAMAG, measurement slit of 6.0 mm × 0.2 mm, for screening 4.0 mm × 0.2 mm) was performed at 500 nm (tungsten halogen lamp, absorbance) after the derivatization with the 2-naphthol sulfuric acid reagent and at 366/> 400 nm (mercury lamp, fluorescence) after the derivatization with the primuline reagent.

### High-resolution mass spectrometry

Steviol glycoside bands (500 ng per 8-mm band each) were marked in the chromatogram with a soft pencil. The respective x-axis coordinates were taken from the application list. The y-axis coordinates were verified by comparison with derivatized reference tracks. The half of the bands (250 ng per 4-mm elution head) were eluted with methanol (flow rate 0.2 mL/min, Dionex Ultimate 3000 UHPLC system, Thermo Fisher Scientific) via the oval elution head [62] (4 mm × 2 mm, TLC-MS Interface 2, CAMAG) and a PEEK inline filter frit (0.5 μm) into the Q Exactive Plus Hybrid Quadrupole-Orbitrap mass spectrometer (Thermo Fisher Scientific). Nitrogen was produced by a SF2 compressor (Atlas Copco Kompressoren and Drucklufttechnik, Essen, Germany). Full scan mass spectra (*m/z* 100–1500) were recorded via heated electrospray ionization (HESI) in the positive and negative ionization mode (HESI spray voltage 3.5 kV, capillary temperature 270 °C, probe heater temperature 200 °C, Sheath/Aux/Sweep gas 20/10/0 AU, maximum injection time 200 ms, lock masses for diisooctyl phthalate at *m/z* 413.26623 and for the formic acid sodium dimer at *m/z* 112.98563). Data were processed via the Xcalibur software (version 3.0.63 with Foundation 3.0 SP2, Thermo Fisher Scientific).

### Bioprofiling and effect-directed detections

Nine chromatograms were prepared in parallel and respective positive controls for each assay were applied bandwise on the upper part of the plate before the assay was performed as described subsequently. All assay solutions were piezoelectrically sprayed (if not stated otherwise, blue nozzle and spraying level 6, Derivatizer, CAMAG). For incubation, the plate was placed horizontally in a humid polypropylene box (26.5 cm × 16 cm × 10 cm, KIS, ABM, Wolframs-Eschenbach, Germany) pre-moistened for 30 min at room temperature (with 35 mL water spread on filter papers aligned on walls and bottom). If not stated otherwise, images were documented under white light illumination (DigiStore Documentation System, CAMAG) after final plate drying (3 min). For the *B. subtilis* and DPPH^•^ assays, the image was captured again after 1 day because the signal response increased.

- For the Gram-negative *A. fischeri* bioassay, the instant green-blue bioluminescence was visually proven by shaking the culture flask (150 μL bacterial cryostock per 20 mL medium according to DIN EN ISO 11348-1 [55], incubated in 100 mL Erlenmeyer flask at 75 rpm and room temperature for 18–24 h) in a dark room. Thereof, 4 mL bacteria suspension was sprayed on the plate. The settling down of the vapor was interrupted to immediately transfer the humid plate to the cabinet of the BioLuminizer (CAMAG). For detection of the antibacterial bands that impaired the instant bioluminescence, 13 images were recorded over 36 min with an exposure time of 60 s and trigger interval of 3.0 min. The positive control (PC) was caffeine (0.5, 1.5, and 3 μL/band, 1 mg/mL in methanol).
- For the Gram-positive *B. subtilis* bioassay, 3.5 mL bacteria suspension (100 μL bacterial cryostock per 20 mL 2.3% Müeller-Hinton broth, optical density of ca. 0.8 at 600 nm [63]) was sprayed, followed by incubation at 37 °C for 2 h. For generation of the colorless (white) antibacterial bands on a purple background, the plate was sprayed with 500 μL 0.2% phosphate-buffered saline (0.8% sodium chloride, 0.02% potassium chloride, 0.14% disodium hydrogen phosphate, and 0.02% potassium dihydrogen phosphate)-buffered MTT solution and incubated at 37 °C for 60 min, followed by drying (50 °C, 5 min, Plate Heater, CAMAG). The PC was tetracycline (0.5, 1.5, and 3 μL/band, 0.004 mg/mL in ethanol).
- For the β-glucuronidase assay [64], 0.5 mL potassium phosphate buffer (0.1 M, pH 7.0, for prewetting) and then 3.0 mL of β-glucuronidase solution (25 U/mL in previous buffer) were sprayed (yellow nozzle), followed by incubation at 37 °C for 15 min. For generation of the colorless (white) inhibition bands on an indigo-blue background, the plate was sprayed (red nozzle) with 1.0 mL 5-bromo-4-chloro-3-indolyl-β-d-glucuronide solution (2 mg/mL in water) and incubated at 37 °C for 1 h. The PC was d-saccharolactone (0.5, 1.5, and 3 μL/band, 0.1 mg/mL in water).
- For the tyrosinase assay, 2 mL each of substrate solution (4.5 mg/mL levodopa in phosphate buffer, plus 2.5 mg CHAPS, and 7.5 mg PEG 8000), and after drying (1 min, stream of cold air, hair dryer) tyrosinase solution (400 U/mL in phosphate buffer, 20 mM, pH 6.8) were sprayed, followed by incubation at room temperature for 15–20 min to generate the colorless (white) inhibition bands on a gray background. The PC was kojic acid (1, 3, and 6 μL/band, 0.1 mg/mL in ethanol).
- For the α-glucosidase assay (yellow nozzle), 2 mL substrate solution (12 mg 2-naphthyl-α-d-glucopyranoside in 9 mL ethanol and 1 mL 10 mM sodium chloride solution) was sprayed as well as after drying (1 min), 1 mL sodium acetate buffer (10.3 g in 250 mL water, add pH 7.5 with 0.1 M acetic acid) and then 2 mL α-glucosidase solution (10 U/mL in sodium acetate buffer, pH 7.5), followed by incubation at 37 °C for 15 min. For generation of the colorless (white) inhibition bands on a purple background, the plate was sprayed with 750 μL aqueous Fast Blue B salt solution (2 mg/mL in water) and dried (3 min). The PC was acarbose (1, 3, and 6 μL/band, 3 mg/mL in ethanol). For the β-glucosidase assay, the enzyme concentration was 1000 U/mL for a 30-min incubation time using 2-naphthyl-β-d-glucopyranoside as substrate. The PC was imidazole (2, 5, and 8 μL/band, 1 mg/mL in ethanol).
- For the AChE assay (green nozzle), 1 mL Tris-HCl buffer (pH 7.8, 0.05 M, for prewetting) and then 3 mL enzyme solution (6.66 U/mL in Tris-HCl buffer plus 1 mg BSA) were sprayed followed by incubation at 37 °C for 25 min. For generation of the colorless (white) inhibition bands on a purple background, the plate was sprayed with 750 μL of a 1:2 mixture of 1-naphthyl acetate (3 mg/mL in ethanol) and Fast Blue B salt solution (3 mg/mL in water) and dried for 2 min. For the butyrylcholinesterase (BChE) assay, the enzyme solution was 3.34 U/mL and the workflow analogously. The PC was rivastigmine (0.5, 1.5, and 3 μL/band, 0.1 mg/mL in methanol).
- For the DPPH^•^ assay, 4 mL 0.04% methanolic DPPH^•^ solution was sprayed (yellow nozzle and level 4). Yellow bands on a purple background were instantly generated. The PC was gallic acid (0.5, 1.5, and 3 μL/band, 0.1 mg/mL in methanol).

## Results and discussion

### Development of the HI-HPTLC-UV/Vis/FLD-HRMS method

**Fig. 1:**
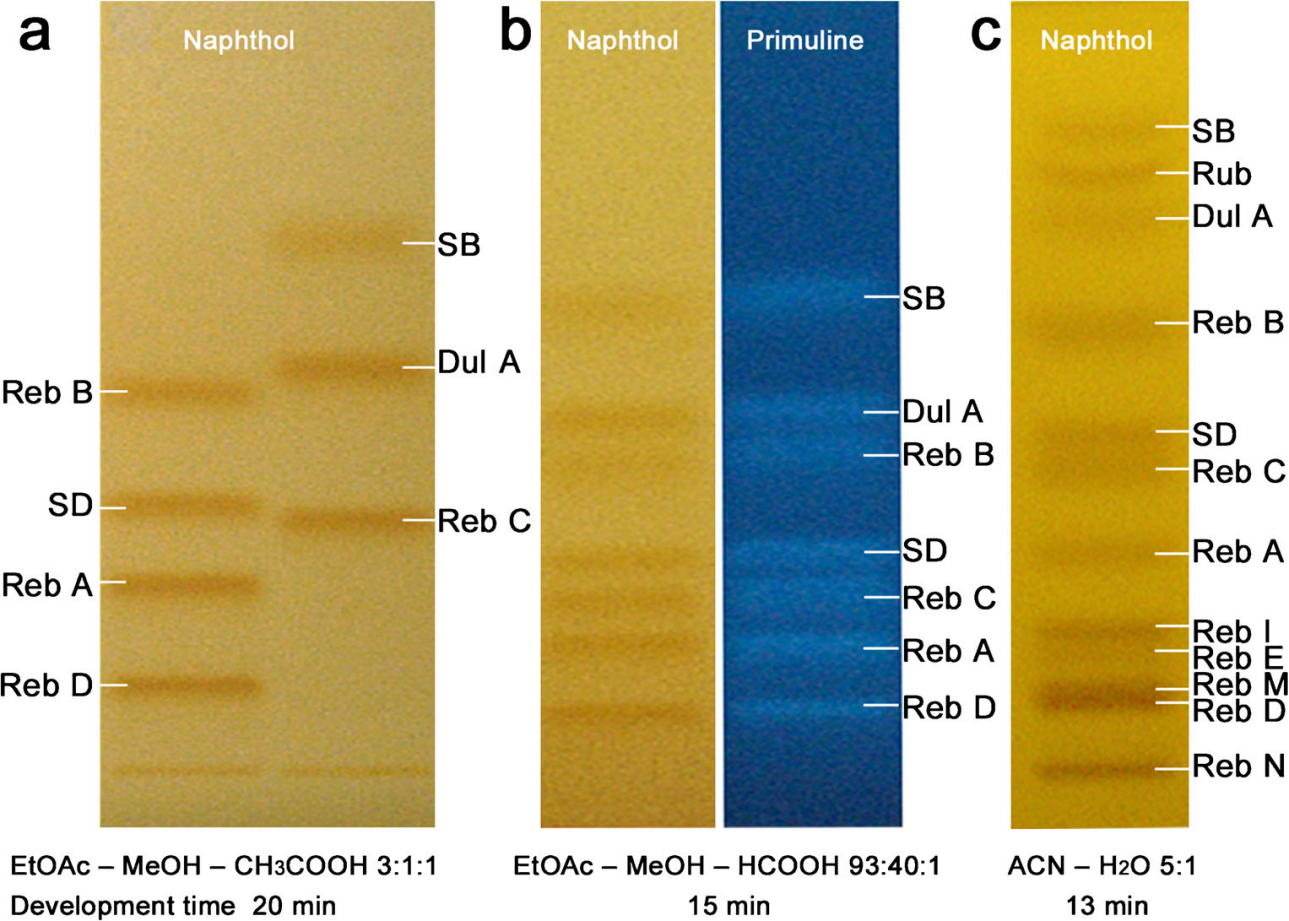
Improvement of the separation methods for steviol glycoside analysis on HPTLC plates silica gel 60 over time: HPTLC-Vis/FLD chromatograms of the previous methods **a** [33] and **b** [5] versus **c** the newly developed HILIC method for 12 steviol glycosides; detected after derivatization either with the 2-naphthol sulfuric acid reagent (60 ng/band [5] or 100 ng/band each) at white light illumination (Vis) or primuline reagent (600 ng/band each) at UV 366 nm (FLD)

The biotechnologically produced steviol glycosides containing more saccharide moieties attract interest as off-flavor-free candidates. Reb M and Reb N, each containing six saccharide moieties, and Reb I and Reb D each containing five, were selected as such representatives of higher molecular weight (ESM Table S2). In contrast to previous normal phase developments on HPTLC plates silica gel 60 (with and without F_254_) with ethyl acetate as selectivity giving solvent and an acid for zone focusing and fine tuning of the selectivity (Fig. 1a [5] and b [33]), HILIC-type separations were explored this time. As HPTLC is a planar form of liquid chromatography, the term hydrophilic interaction high-performance thin-layer chromatography (HI-HPTLC) was coined. Different mixtures of acetonitrile with water or ammonium acetate buffer (50 mM) were studied. The *hR*_F_ values of the individual steviol glycosides as well as the developing times increased with the water ratio of the mobile phase mixture. The ammonium acetate buffer did not generate a better zone resolution when compared with water. The addition of 2-aminoethyl diphenylborinate or boric acid that usually improves the resolution of glucose and fructose [65] was also investigated. However, it did not generate a benefit for the separation of steviol glycosides, except for slightly increasing *hR*_F_ values with increasing additions (ESM Fig. S1). Hence, acetonitrile-water mixtures (5:1 to 4:1, V/V) were preferred taking 13 min up to 60 mm in the twin trough chamber, and 10 min up to 50 mm in the horizontal development chamber. This was found to be a fast option for later screening of food samples.

The steviol glycosides were separated according to their solubility/partitioning, polarity, hydrophilic interaction (*hR*_F_ values increased with decreasing saccharide moieties almost analogously﻿, ESM Table S2), and steric orientation (saccharides at both R1/R2 positions or at the same R position). All 12 steviol glycosides were separated between *hR*_F_ 6 and 83 (Fig. 1c). Steviol and isosteviol were located at *hR*_F_ > 95 (before the solvent front, detectable with the primuline or anisaldehyde sulfuric acid reagents). When comparing three different saccharide-selective reagents for derivatization of the steviol glycosides, i.e., the *p*-aminobenzoic acid reagent, diphenylamine aniline phosphoric acid reagent, and 2-naphthol sulfuric acid reagent (ESM Fig. S2, prepared as reported [56–58]), the latter was most sensitively detecting the steviol glycosides. However, the primuline reagent and also the anisaldehyde sulfuric acid reagent detected additional steviol and isosteviol, which was advantageous as discussed later.

**Fig. 2:**
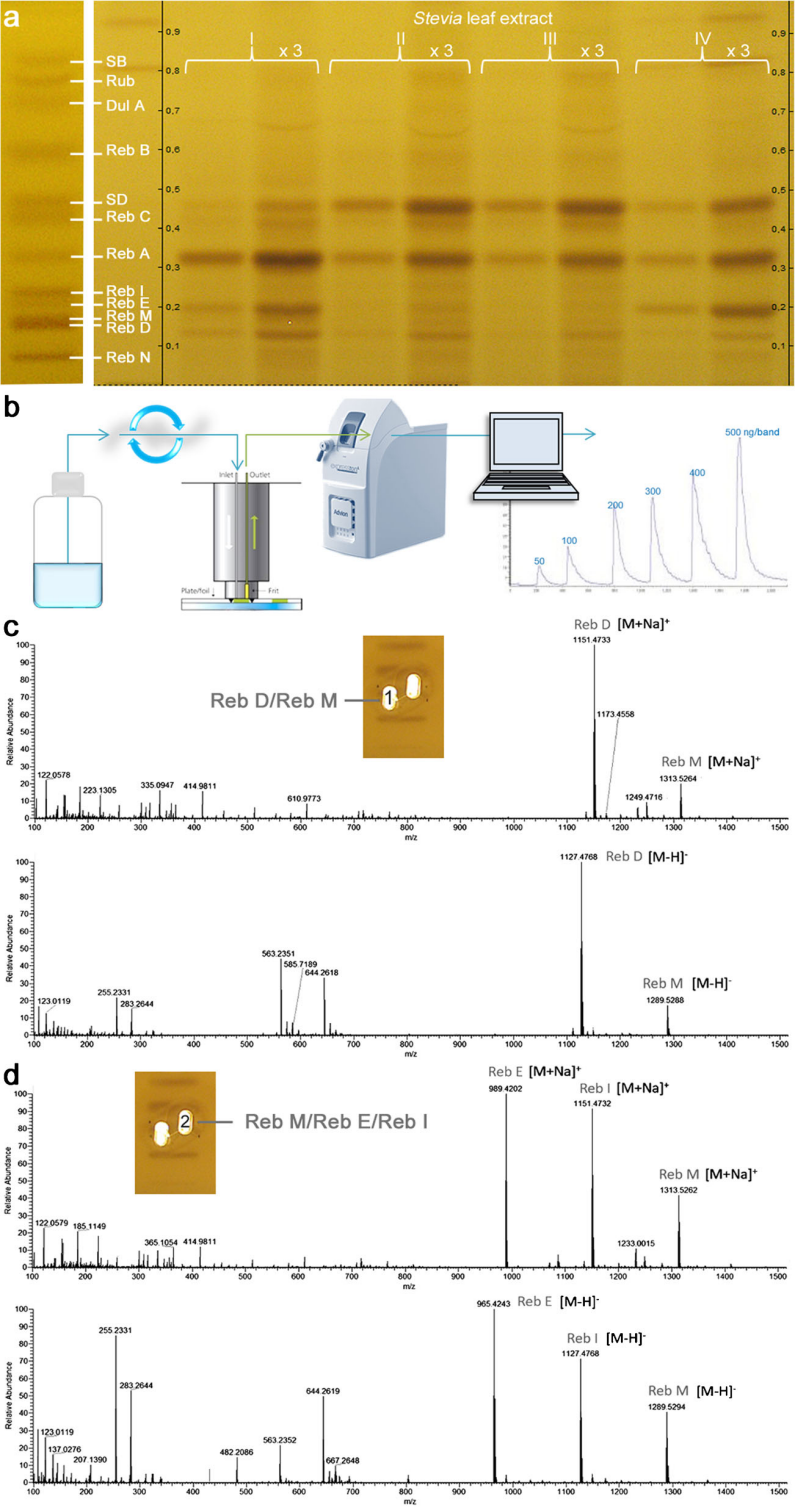
*Stevia* leaf analysis. **a** HI-HPTLC-Vis chromatogram of *Stevia* leaf samples I-IV (extracted with ethanol-water 4:1; 10 mg/mL each; 2 and 6 μL/band each) on HPTLC plate silica gel 60 F_254_ with acetonitrile-water 5:1 and detected after derivatization with the 2-naphthol sulfuric acid reagent at white light illumination. **b** Scheme for recording of HPTLC-MS spectra via an elution head-based interface. **c** The plate turned by 90° for zone elution (elution head positioned vertical to catch two bands) proved that a coeluting critical pair would clearly be noticed by the characteristic mass signals in the HI-HPTLC-HESI-HRMS spectra, both in the positive and negative ion mode; the post-MS derivatization image shows the respective elution head imprints 1 and 2

**Fig. 3:**
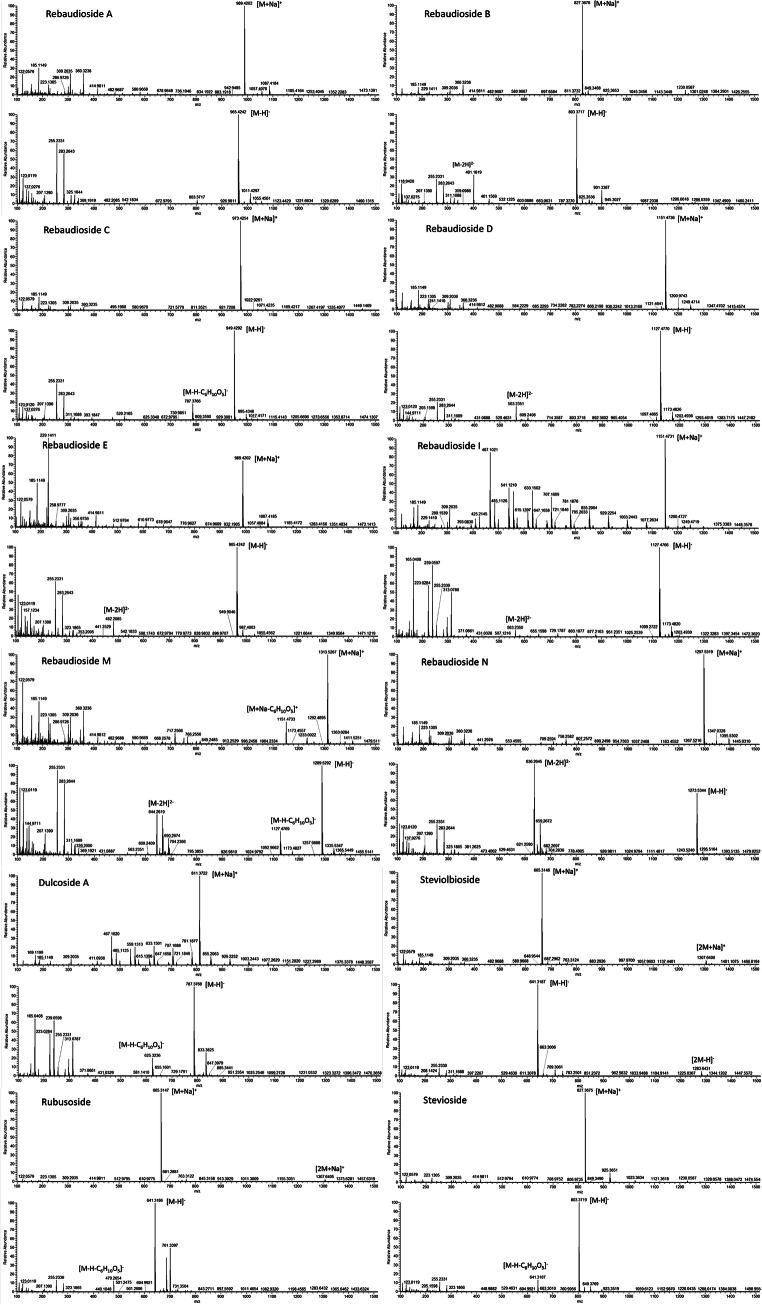
HI-HPTLC-HESI-HRMS mass spectra (*m*/*z* 100 to 1500) after separation with acetonitrile-water 5:1 on prewashed HPTLC plate silica gel 60 F_254_: the respective deprotonated molecule [M-H]^−^ in the negative HESI mode and its sodium adduct [M + Na]^+^ in the positive HESI mode were obtained as base peaks for each of the 12 steviol glycosides (250 ng/elution each)

A baseline separation of all 12 steviol glycosides was not obtainable (e.g., critical pair of Reb M/D, Fig. 1c). Nevertheless, when applied to the *Stevia* leaf analysis, the resulting steviol glycoside band pattern was clearly resolved, as not all steviol glycosides are present at the same time in a sample at such relevant high amounts (Fig. 2a). When a potential coelution is assumed, an HPTLC-HRMS spectrum can be recorded to figure out the steviol glycoside present (Fig. 2b). In the mass spectrum, this would be noticed by the characteristic mass signals. A coelution was simulated by a 90° plate turn for zone elution with the oval elution head, and thus eluting two adjacent steviol glycoside bands at one go. As evident in the resulting mass spectra (Fig. 2c, d), HPTLC-HRMS was proven to distinguish mass-selectively the individual steviol glycosides eluted. For the first time, HI-HPTLC-HESI-HRMS spectra were recorded for the 12 steviol glycoside bands (ESM Fig. S3a, b). For each steviol glycoside (250 ng/elution each), a pronounced base peak was obtained in the full scan mass spectrum (*m*/*z* 100–1500, Fig. 3, ESM Table S2). It was either the respective deprotonated molecule [M-H]^−^ in the negative HESI mode or its sodium adduct [M + Na]^+^ in the positive HESI mode. The mean mass error of the measured versus theoretical *m/z* of the steviol glycosides was − 0.78 ppm (between − 0.04 and − 1.54 ppm). Isobaric masses in HRMS (Reb A/E or Reb I/D) were separated by HPTLC and clearly distinguished by HPTLC-HRMS. Fragmentation patterns were dedicatedly studied elsewhere [27]. In the negative ionization mode, the repeated detection of the ion signals at *m/z* 255.2331 and 283.2643 especially in most Reb spectra due to the lower signal response and thus reduced S/N (Fig. 3) was assigned as palmitic and stearic acids, respectively, and caused by the disposable gloves used. This was easily figured out, as these ions were also detected in background spectra and respective extracted ion current chronograms (ESM Fig. S3c, d). Hence, powder-free gloves are relevant not only for a good laboratory practice in HRMS recording [60] but also for the whole HPTLC-HRMS procedure to avoid this background contamination.

### Development of the bioactivity profiling

*Stevia* leaf extracts were reported to contain miscellaneous minor compounds other than steviol glycosides [66, 67]. Instead of adding the chemically isolated, purified, and EU-regulated steviol glycosides to food, the addition and consumption of the more natural leaf extracts or the leaves themselves might have benefits for health. Though *Stevia* leaves were used in traditional medicines for diverse purposes [66], effect-directed in vitro assays have hardly been performed so far [67, 68]. In order to obtain more information on the individual bioactivities of *Stevia* leaf extracts versus steviol glycosides, a non-targeted bioprofiling was developed. This bioanalytical technique consisted of a chromatographic separation and a biological or biochemical detection. Four *Stevia* leave products were extracted with ethanol-water 4:1 as well as ethyl acetate for their effect-directed analysis. Each was prepared as a 1% suspension (ESM Table S1), which was found to be a sufficiently high concentration to start with the effect-directed analysis. The mobile phase of acetonitrile-water was perfectly suited for application of the different assays, as it contained no acid or base.

After the HILIC separation, the chemical profiles of the four *Stevia* leaf extracts pointed to red fluorescent chlorophylls and blue fluorescent phenolic compounds being present (ESM Fig. S4a–c). The latter were confirmed to be flavonoids by derivatization of the same chromatogram with the natural product reagent (ESM Fig. S4d [59]). Instead of immersion [5], piezoelectric spraying was exploited and optimized for all applied derivatization reagents regarding spray volume, nozzle, and level. Another chromatogram was subjected to the universally detecting anisaldehyde sulfuric acid reagent; the steviol glycosides turned green and yellow (ESM Fig. S4e). The individual steviol glycoside colors were investigated in detail (ESM Fig. S5). The three rhamnose containing glycosides (Reb N, Reb C, and Dul A) turned yellow by this derivatization on the HPTLC plate silica gel 60 F_254_ after heating at 110 °C for 5 min. This color did change to olive green for a prolonged heating period or on plates without fluorescence indicator (ESM Fig. S5 a versus c). Especially the steviol glycosides of critical pairs were colored green and yellow. This was found to be helpful, as coeluting compounds would be indicated by a yellow halo effect, in contrast to the zones of the same brown hue obtained after derivatization with the 2-naphthol sulfuric acid reagent (ESM Fig. S4f). By these physicochemical characterizations via multi-imaging (six detection modes performed on three plates, ESM Fig. S4), diverse information on the leaf samples was collected. All observed compounds were spread along the developing distance, which was a good precondition to proceed with the biological or biochemical detection.

**Fig. 4:**
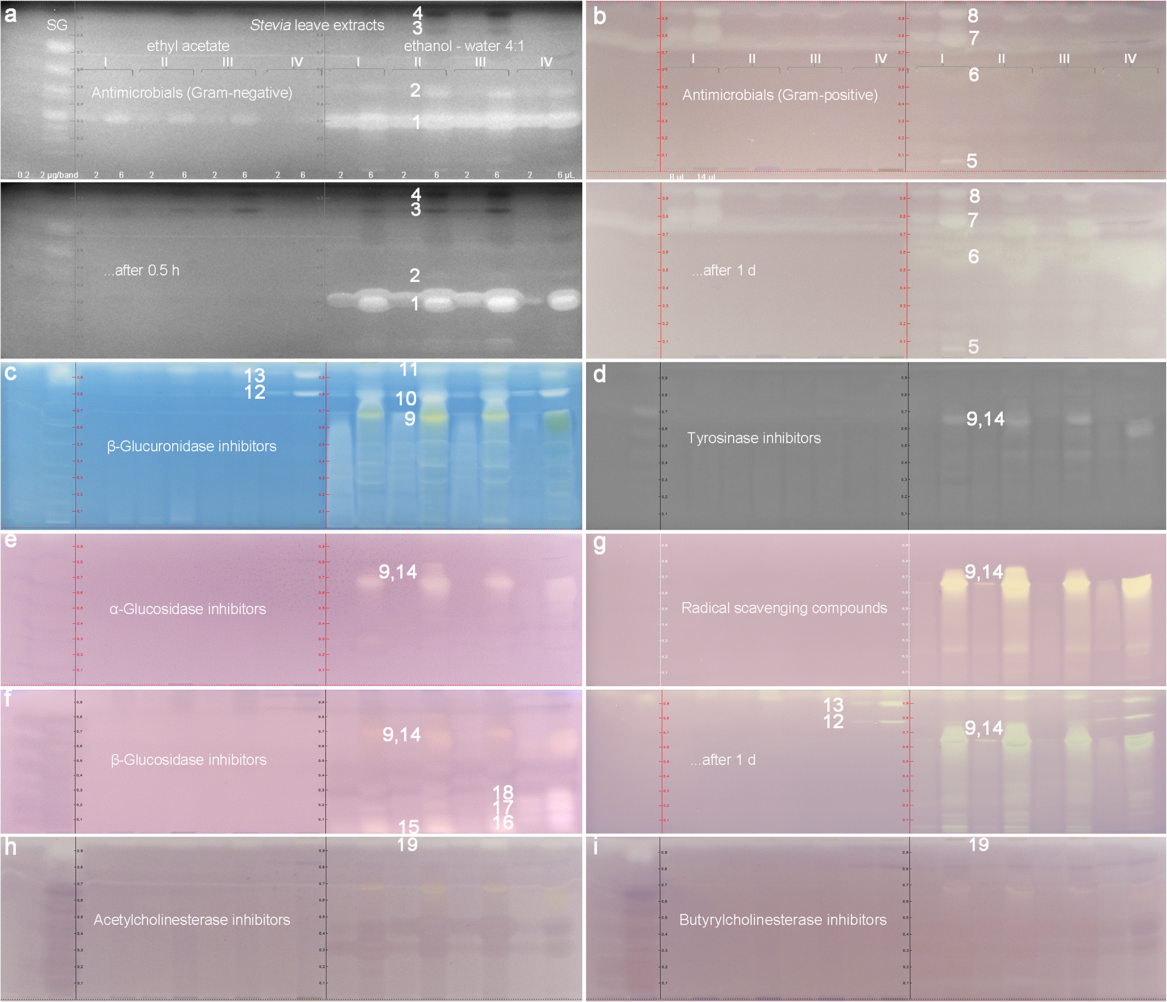
Bioprofiling of steviol glycosides (SG; 0.2 and 2 μg/band) and *Stevia* leaf samples I–IV extracted either with ethyl acetate or ethanol-water 4:1 (10 mg/mL each, 2 and 6 μL/band each): HI-HPTLC (bio) autograms showing 19 bioactive bands found after the **a** antimicrobial Gram-negative *A. fischeri* (bioluminescence depicted as greyscale instantly and after 27 min), **b** Gram-positive *B. subtilis* (higher volume applied, 8 and 14 μL/band each; detected also after 1 day), **c** β-glucuronidase, **d** tyrosinase, **e** α-glucosidase, **f** β-glucosidase, **g** DPPH^•^ (detected also after 1 day), **h** AChE, and **i** BChE assays. **b**–**i** Documented at white light illumination

Enzyme solutions and bacteria suspensions were piezoelectrically sprayed on the plate for the first time for all nine assays. After an initial investigation [69], the workflow was streamlined and optimized with regard to prewetting, spray volume, spray nozzle, spray level, intermediate drying, and positive controls. Nine chromatograms were prepared analogously and subjected to the nine different effect-directed assays (Fig. 4). These detected antibacterials (i.e., against Gram-negative *A. fischeri* and Gram-positive *B. subtilis* bacteria), various enzyme inhibitors (i.e., against tyrosinase, α-glucosidase, β-glucosidase, AChE, BChE, and β-glucuronidase), and radical scavenging compounds (DPPH^•^ assay). As proof of the proper performance of each assay, a respective positive control was selected according to importance, significance, and costs. Before the assay application, each positive control was applied bandwise at three different volumes and thus amounts on the upper plate edge (ESM Fig. S6). This general practice was found to be efficient because it did not afford an extra track and optimization of separations, but clearly proved the proper performance of the assay. The proper color formation on the plate background was considered as negative control. A blank of each extraction solvent was applied as control on an edge track, but did not contribute to the effect-directed signals discussed later. Information on a potential side reaction of the chromogenic substrate other than that with the enzyme can also be obtained. This was exemplarily performed for the chromogenic substrate Fast Blue B salt solution used in the α-glucosidase, β-glucosidase, AChE, and BChE assays. Any side reaction was not observable (ESM Fig. S7).

For all assays, 20 and 60 μg of each *Stevia* leaf extract were loaded on the start band, except for the *B. subtilis* bioassay (80 and 140 μg needed to detect an effect). Of course, if higher amounts are applied, further weaker active components can be detected, but this was considered to be of minor importance. For comparison, the steviol glycoside mixture was applied at two different volumes and thus amounts of 0.2 and 2 μg/band to imitate a comparable low and high steviol glycoside content of the leaves, as proven in the chromatogram after derivatization with the 2-naphthol sulfuric acid reagent (ESM Fig. S4f). The individual effects observed are discussed in the following.

### Antibacterial or prebiotic compound bands 1 to 8 found in *Stevia* leaf extracts

**Fig. 5:**
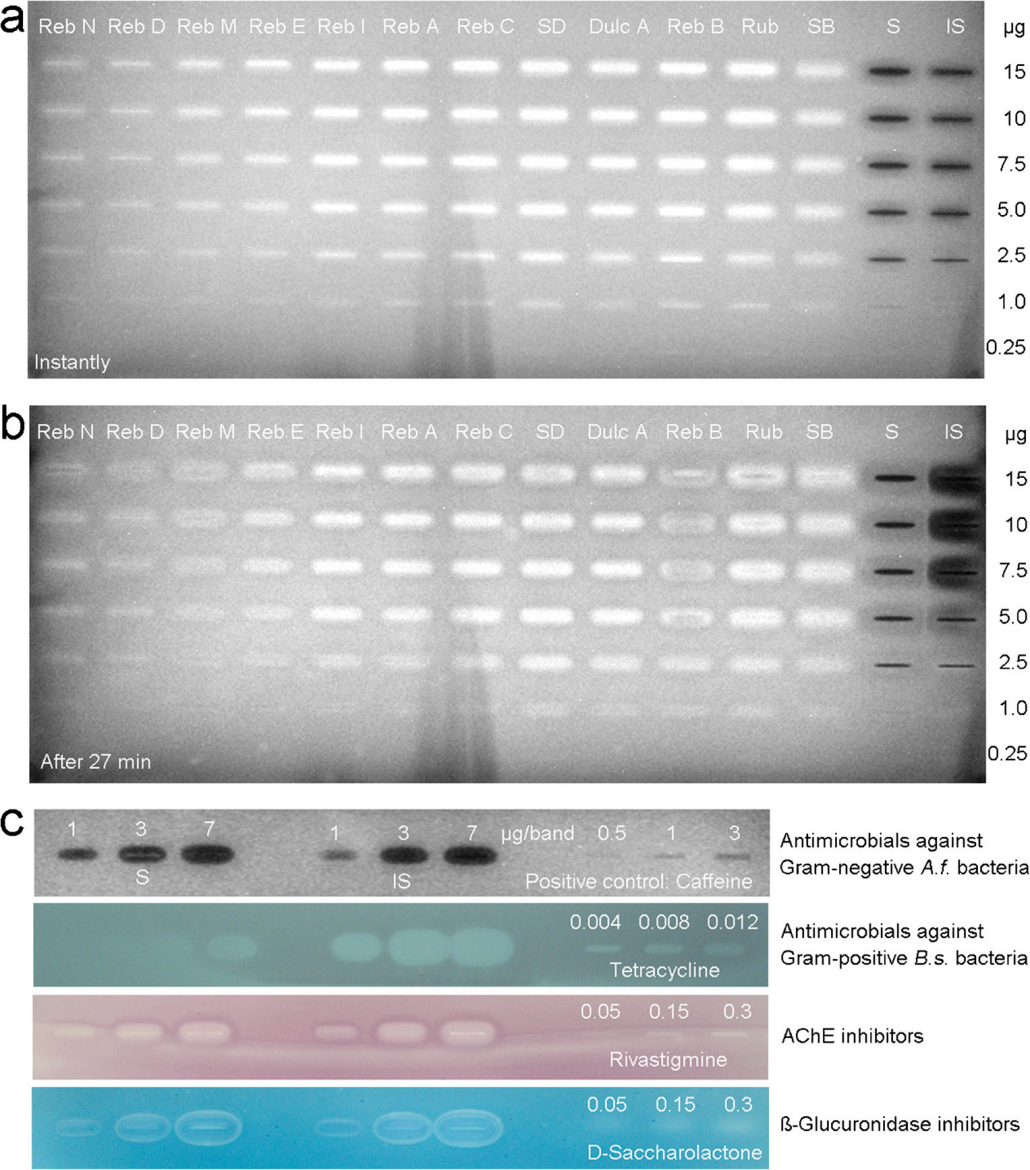
Dose-dependent Gram-negative *A. fischeri* bioluminescence of each steviol glycoside, steviol, and isosteviol (each as 0.25–15 μg/band pattern) applied on HPTLC plate silica gel 60 F_254_ and detected **a** instantly and **b** after 27 min (depicted as greyscale image) as well as **c** detailed bioactivity study on steviol and isosteviol (applied as 1, 3, and 7 μg/band pattern) in comparison with positive controls of the respective Gram-negative *A. fischeri*, Gram-positive *B. subtilis*, AChE and β-glucuronidase assays

In the Gram-negative *A. fischeri* bioautogram, a bioluminescence enhancing band was prominently observed in all ethanol-water extracts, indicating a prebiotic effect (Fig. 4a). In the ethyl acetate extracts, this effect was also observed, although much less pronounced. This strong, bioactive compound band 1 (*hR*_F_ 30) instantly enhanced the bioluminescence metabolism of the bacteria and held on until the last image was recorded after half an hour, although it changed its shape (distinct halo effect for the respective higher amount applied). This active compound band was at the same *hR*_F_ value as rebaudioside A. A further enhancing, yet much weaker band 2 (*hR*_F_ 48) was located at the same *hR*_F_ value as stevioside. In order to study in detail the bioluminescence enhancing effect of each steviol glycoside, a concentration-dependent track pattern (ranged 1:60) of each steviol glycoside as well as steviol and isosteviol was applied (Fig. 5a, b). All steviol glycosides showed a bioluminescence enhancing effect, and thus, delivered more energy for the Gram-negative bacterial communication, although the individual steviol glycosides were differently pronounced. The effect was increasing with decreasing saccharide moieties, except for steviolbioside. Steviol and isosteviol had a strong opposite, antimicrobial effect (reduced bioluminescence and thus reduced bacterial communication). All effects were long lasting (observed for half an hour). Moreover, two comparatively weaker, dark, and thus, antimicrobial bands 3 and 4 were evident at *hR*_F_ 82 and 93 in the leaf extracts II and III. In contrast, saccharides were proven not to be bioactive, as discussed for food analysis.

In the Gram-positive *B. subtilis* bioautogram, an effect was first observed after the application of higher volumes and thus amounts of the leaf extracts (Fig. 4b, 80 and 140 μg/start band each, by a factor of 4 and 2.3 more, respectively) when compared with all other assays performed. Especially in the ethanol-water extract I, four pronounced colorless antimicrobial bands 5–8 were evident at *hR*_F_ 5, 60, 80, and 95, respectively. The antimicrobial bands 7 and 8 were partially also observed in the ethyl acetate extracts. The same autogram revealed that the antimicrobial band **6** increased in its effect over the time (comparatively slower antimicrobial reaction), which was evident when it was documented again on the following day. It became also very diffuse (ranged *hR*_F_ 50–60), especially pronounced in the ethanol-water extract IV, indicating a comparatively higher diffusion coefficient or vapor pressure of this compound. All steviol glycosides showed no effect; however, the potential breakdown products steviol and isosteviol had a strong antimicrobial effect against Gram-positive *B. subtilis* bacteria (Fig. 5c).

### Enzyme-inhibiting compound bands 9 to 19 found in *Stevia* leaf extracts

In the β-glucuronidase assay (Fig. 4c), the diffuse, tailing, colorless inhibition area 9 (*hR*_F_ 0–70) was most likely caused by flavonoids, as it showed a similarly tailing profile as after derivatization with the natural product reagent (ESM Fig. S4d, an acidic instead of neutral mobile phase would reduce this tailing effect). This band 9 was coeluting with the greenish band 14 (discussed later, slightly better separated in extract IV). Two further, intense β-glucuronidase inhibiting bands 10 and 11 (*hR*_F_ 80 and 95, respectively) were observed in the extracts I–III. As one of the rare effects observed in the ethyl acetate extracts, the inhibiting bands 12 and 13 (*hR*_F_ 81 and 92) were evident in extract IV. As the β-glucuronidase inhibiting band 11 was near the solvent front, another development was performed using a less eluting mobile phase. For this experiment (ESM Fig. S8a), steviol and isosteviol were oversprayed on the steviol glycoside mixture (remaining at the start band) and additionally applied as band pattern. As a result, the β-glucuronidase inhibition of band 11 was confirmed, which, however, was now resolved in three bands (ESM Fig. S8a, bands 11a–c). Steviol and isosteviol also inhibited the β-glucuronidase (Fig. 5c). Although the inhibition bands 11a–c were located at comparable *hR*_F_ values to steviol and isosteviol, the band shapes were different. For further clarification, one extract, containing band 11, can be applied overlapped with steviol/isosteviol to exclude a matrix effect that causes this difference in the zone shapes.

In the tyrosinase assay (Fig. 4d), the intense inhibiting band 14 (*hR*_F_ 65, in extract IV at *hR*_F_ 60) was detected in the ethanol-water extracts, coeluting with the comparatively less active band 9. The same active zone pair was also inhibiting the α-glucosidase (Fig. 4e) and β-glucosidase (Fig. 4f). Four further inhibiting compound bands 15–18 were observed in the β-glucosidase assay. In the AChE/BChE assays (Fig. 4h, i), the inhibiting band 19 was evident in the ethanol-water extract III in the solvent front. Again, an analogous separation with a mobile phase mixture of a reduced elution power confirmed this inhibition band at a comparable *hR*_F_ value and zone shape as steviol (ESM Fig. S8b). Steviol and isosteviol were proven to strongly inhibit the AChE/BChE (Fig. 5c), indicating a neurotoxic effect. However, further investigations (overlapped application or recording of mass spectra) are needed to identify the inhibiting band 19.

An additional zone in the front of the steviol glycoside mixture, which was applied at a higher amount (ESM Fig. S8), also inhibited β-glucuronidase and AChE. Using this more apolar mobile phase, its band shape was even and not distorted as noticed in the previous separations of a higher elution power (Fig. 4 versus ESM Fig. S8). This zone was called impurity because it was assumed to be caused by a breakdown or insufficient purity of the steviol glycosides, but can still be a contaminant. The source of this apolar bioactive compound in the steviol glycoside mixture is presently investigated.

### Antioxidative compound bands 9 and 12 to 14 found in *Stevia* leaf extracts

In the DPPH^•^ radical scavenging assay (Fig. 4g), the same tyrosinase/α-glucosidase/β-glucosidase inhibiting zone pair of 9 and 14 was most pronounced. The previously reported β-glucuronidase inhibiting compounds 12 and 13 in the extract IV were also detectable when documented after 1 day (same pattern). This delayed expression of the radical scavenging effect indicates either a comparatively slower mechanism of the radical reaction or a precursor affording first an oxidative activation (by the open planar system).

### Outcome of the comparative bioprofiling

The bioprofiling as an imaging technique illustrates well the benefits of the intake of natural leaf products over that of chemically isolated and purified steviol glycosides. We figured out that *Stevia* leaf extracts do affect the activity of microbials, α-glucosidase, β-glucosidase, β-glucuronidase, AChE, BChE, tyrosinase as well as radicals present. Multipotent multicomponent botanical mixtures may have subtle impact on homeostasis via several metabolic pathways. The more natural leaf extracts contained up to 19 different bioactive compounds, whereas the steviol glycosides (applied at comparable amounts) showed only a prebiotic effect for the selected Gram-negative bacteria (Fig. 4a) and a comparatively weak tyrosinase inhibition (Fig. 4d). Upon degradation of steviol glycosides to steviol and isosteviol, for example, by critical processing steps or storage of *Stevia* leaf products, these degradation products can much more stronger inhibit AChE, BChE, β-glucuronidase, Gram-negative *A. fischeri*, and Gram-positive *B. subtilis* bacteria (Fig. 5c). With regard to the tested effects, these 19 bioactive bands represent the most active compounds predominantly found in the ethanol-water extracts of the investigated *Stevia* leaves. The zone pair of 9 and 14 turned out to be multipotent (same pattern was evident), being an inhibitor of tyrosinase, α-glucosidase, and β-glucosidase as well as a radical scavenger. Band 9 additionally inhibited the β-glucuronidase. Bands 12 and 13 in leaf extract IV were also active as β-glucuronidase inhibitors and radical scavengers. All nine assays revealed bioactive compounds present, among which the detected antimicrobials against Gram-positive *B. subtilis* bacteria were not so pronounced, as a comparatively higher application volume of the leave extracts was required to obtain a response. The total analysis time for 20 samples in parallel depended on the assay (between 1 h and 4 h). The instantly bioluminescent *A. fischeri* bioassay or the DPPH^•^ radical scavenging assay were fastest and took 3 min/sample, whereas due to the long incubation time (3 h), the *B. subtilis* bioassay took 12 min/sample. Enzyme assays took 5–7 min/sample depending on the total incubation time. Running costs are about 0.5 Euro/sample. For routine bioprofilings, the choice of an assay depends on what information about an effect is desired for quality control and safety of products. In particular, the detection of estrogen-like, androgen-like, genotoxic, and agonistic/antagonistic effects, which have been developed recently [46], should be of interest. As demonstrated in previous studies, the individual active compounds can further be characterized by HPTLC-HESI-HRMS and elucidated in its structure [64, 70]. Quantification of the discovered activities can be performed by external standards or a well-known reference (equivalency calculation), when a standard compound is not available or the bioactive compound is unknown/unidentified [63, 71].

### Analysis of 20 different food samples containing steviol glycosides

One cereal porridge mixture, two tea mixtures, six powders, six crystal granulates, four tablet products, and one liquid extract (aqueous fluid) were exemplarily investigated as food samples containing steviol glycosides (ESM Table S1). The extraction of steviol glycosides from food had to respect the different solubility of the individual steviol glycosides in aqueous and organic solvents. Water was needed for the reason of solubility. Methanol was preferred to ethanol, as methanolic solutions are faster sprayed on the HPTLC plate. Hence, a good compromise was found to be an extracting solvent of methanol-water 9:1. The sample preparation was carried out in a minimalistic way with regard to the different matrices. Altogether, it took 3.5 h for the 20 different products (ESM Table S1). Up to 48 analyses were performed via a horizontal antiparallel development of the HPTLC plate. The separation took 10 min (calculated to be 13 s/sample) and consumed 8 mL mobile phase mixture (4 mL per plate side or 0.17 mL/sample). Quantification was best performed via a 4-point calibration in the range of 34 to 340 ng/band (0.4–4 μL standard mixture applied) and absorbance measurement at 500 nm after derivatization with the 2-naphthol sulfuric acid reagent. Hence, the total twofold analysis of 20 food samples took less than 5 h including sample preparation (7.5 min/analysis). The running costs of such a screening summed up to 0.3 Euro per analysis.

**Fig. 6:**
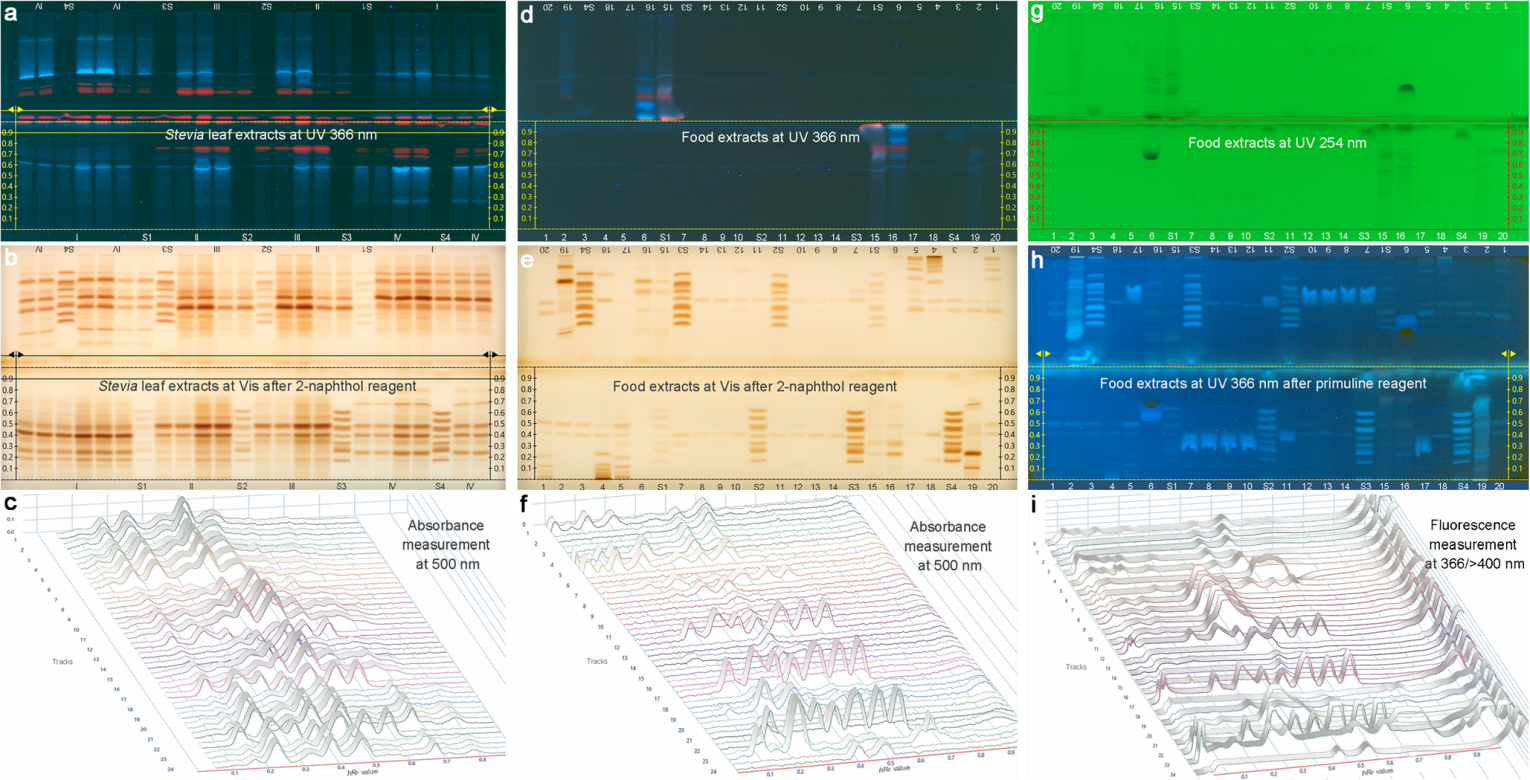
Antiparallel development in the horizontal developing chamber taking 10 min on the HPTLC plate silica gel 60 F_254_ with 8 mL acetonitrile-water 4:1 (4 mL per plate side): HI-HPTLC-Vis/FLD chromatograms of **a–c** *Stevia* leaf extracts (1.5 and 3 μL/6 mm-band each) and **d–i** 20 different food products containing steviol glycosides (ID assignment in ESM Table S1; twofold determination) along with steviol glycoside calibration levels (S1–S4; 1–10 μL/6 mm-band each), detected **a**, and **d** at UV 366 nm, **g** at UV 254 nm and after derivatization with 2-naphthol sulfuric acid reagent **b**, and **e** at white light illumination; **g–i** same, but 7-fold higher volumes applied and detected by the primuline reagent at UV 366 nm; **c**, **f**, and **i** respective densitograms of one plate half recorded at 500 nm or 366/> 400 nm

Exemplarily, an antiparallel development and the respective densitometric scan at 500 nm is demonstrated for the *Stevia* leave extracts (Fig. 6a–c) and food products (Fig. 6d–f). In one sample (ID 6, ESM Table S1), steviol glycosides were not detectable. As it tasted sweet, it was investigated for other sweeteners. Instead of any steviol glycoside as labelled, the sample contained sodium cyclamate and saccharine (ESM Fig. S9). Among the co-extracting polar compounds, saccharides or derivatives other than steviol glycosides may interfere, all detectable with this saccharide-selective derivatization reagent. Hence, 19 different saccharides and 5 different types of derivatives (such as sugar alcohols, glycosyl amines, its acetylated form, glycosyl phosphates, or glycuronic acids) were separated using the new HILIC system to figure out any interference (ESM Fig. S10a). As the main saccharide in food, sucrose did not interfere because it was located above the *hR*_F_ range of all the steviol glycosides. Fructose, glucose, galactose, and lactose eluted in the *hR*_F_ range of Reb I, Reb E, and Reb N, respectively. This was found to be acceptable, as the latter three have hardly been added as sweetener to food products up to now.

The *A. f.* bioassay was also applied for the 19 different saccharides and 5 different types of derivatives. In contrast to the prebiotic effect observed for the steviol glycosides (Fig. 5c), it proved that these 24 saccharides were not bioactive, even not for application as high as 2 μg/band (ESM Fig. S10b). However, steviol and isosteviol showed an antibacterial activity that was stronger than that of the positive control caffeine (comparison of the 1 μg/band applications in ESM Fig. S10b).

Nevertheless, in case of saccharide interferences, the use of the highly selective primuline reagent followed by fluorescence measurement at 366/> 400 nm is superior, as it detects vice versa the steviol aglycon site instead of the saccharide moiety. It physisorbs to the apolar site and detects steviol glycosides as blue fluorescence, but not saccharides and most derivatives in case of being present. Hence, as an example, the same analysis was performed using the primuline reagent (Fig. 6g–i). The primuline reagent detects the steviol glycosides less sensitive and the applied volumes needed to be multiplied by 7. As a benefit, this reagent is compatible to direct HPTLC-HRMS recording, as it does not change the mass signals in the mass spectra recorded. In the ion source, the weakly physisorbed primuline easily separates from the steviol glycosides and is detected separately. A blue fluorescent band deformation was observed for some samples (ID 7 to 10 and 17) that interfered also with Reb A. This was observed first at the 7-fold higher volume applied (Fig. 6h versus e). In common, these five samples contained not only rebaudioside A, but also erythrit. This was also true for the erythrit-containing sample ID 11, which had a higher sweetening power. However, its lower erythrit content did not cause this interference, but it was detected now as additional blue fluorescent compound (not distorted). Hence, a higher erythrit content caused this band interference, and higher erythrit additions were easily detectable as such. Nevertheless, the blue fluorescent interference was not caused by erythrit itself, because primuline does not detect erythrit. It can be a lipophilic additive in erythrit-containing formulations, which was not clear by the given labelling. Not even showing a single steviol glycoside, the falsified sample ID 6 (ESM Table S1) was easily discovered by this reagent, too.

### 2-Step separation of steviol, isosteviol, and steviol glycosides

The joint detection of steviol glycosides, steviol, and isosteviol as blue fluorescent bands at UV 366 nm by the primuline reagent (physisorption to the lipophilic moiety) was investigated in our previous HPTLC study [5]. The plate developed up to 60 mm was cut in the plate middle. The lower plate part containing the steviol glycosides was detected by the 2-naphthol sulfuric acid reagent. The upper plate part containing steviol and isosteviol (near the solvent front) was developed and detected with the primuline reagent (ESM Fig. S11). This 2-step approach for baseline separation of steviol and isosteviol was transferred to the new HILIC-type separation. Steviol and isosteviol also eluted with the solvent front with the more polar HILIC solvents. However, their band shapes were distorted by an apolar impurity present in the steviol glycoside mixture (Fig. 6e, tracks S3 and S4, better visible in Fig. 6h via the primuline reagent-detecting lipophilic compounds). This was proven by another development of steviol and isosteviol on separate tracks, for which the band shape was good. The impurity was a lipophilic bioactive (inhibited β-glucuronidase and AChE) compound in the standard mixture which was distorted during migration in the polar mobile phase used for the steviol glycoside separation and impaired the steviol and isosteviol bands. As expected, in a more apolar mobile phase, the band shape of the impurity was even, but steviol glycosides were not resolved (ESM Fig. S8, remained at the start zone).

**Fig. 7:**
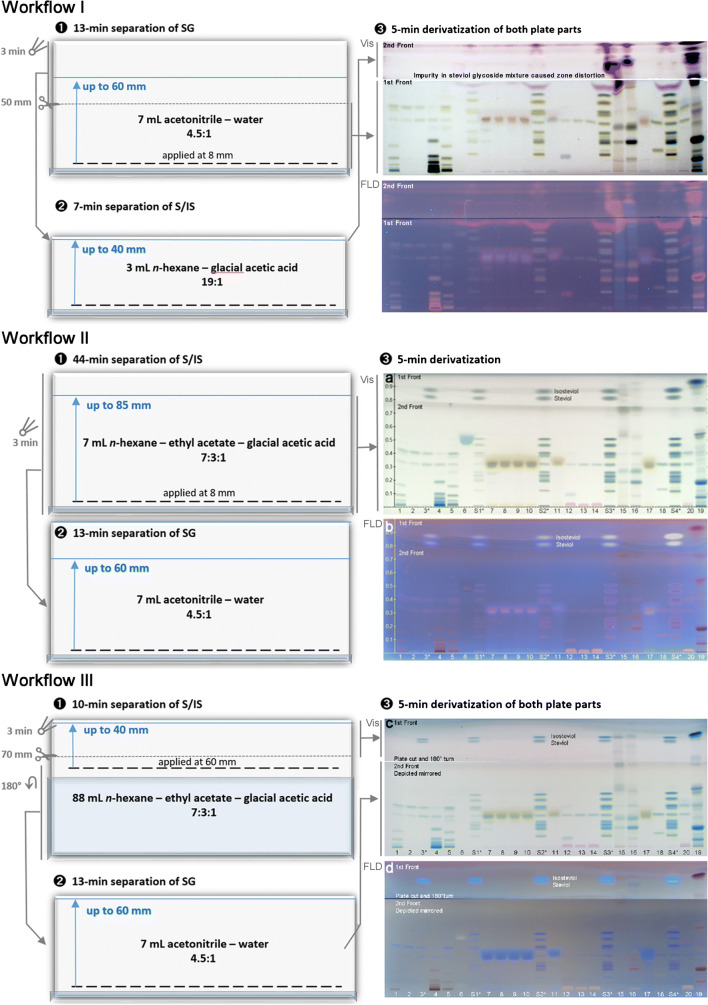
Three options for 2-step separation of steviol, isosteviol, and steviol glycosides: workflow I (20 min analysis time, 10 mL solvent consumption, but impaired by an impurity) versus workflow II (60 min analysis time, 14 mL solvent consumption) versus workflow III (20 min analysis time, 95 mL solvent consumption); after workflow I, some sample volumes were slightly reduced; ID assignment in ESM Table S1 (same as Fig. 6), but overspray of steviol and isosteviol on S1–S4 (1–6 μg/band, marked*), and exemplarily as proof, on ID 3 (2 μg/band); detection at white light illumination (Vis) and UV 366 nm (FLD) after anisaldehyde sulfuric acid reagent

Since the band impairment increased with increasing amounts of the steviol glycoside mixture on the plate, a more sensitive derivatization reagent was considered. Analogous to the primuline reagent, steviol, isosteviol, and steviol glycosides can be detected together via the anisaldehyde sulfuric acid reagent. The latter was more sensitive than the primuline reagent, i.e., needed only the 4-fold (anisaldehyde sulfuric acid reagent) instead of 7-fold application volumes (primuline reagent) when compared with 2-naphthol sulfuric acid reagent as reference. First when heated at a higher temperature (160 °C instead of 110 °C), steviol and isosteviol were detectable as blue-colored band. The additional sensitive detection of steviol and isosteviol as blue fluorescent bands was advantageously (Fig. 7). Also with this reagent, the falsified sample ID 6 was eye-catching und directly discovered. The six erythrit-containing samples (ID 7 to 11 and 17) that interfered with rebaudioside A were easily detected as such because the more diffuse erythrit zone was derivatized to a pink (heated 110 °C, workflow I in Fig. 7) or yellow zone (160 °C, workflow II and III in Fig. 7) when using the anisaldehyde sulfuric acid reagent. As the temperature of the derivatization reaction had a visible influence on the color formation of erythrit (pink or yellow) and rhamnose-containing steviol glycosides (yellow or olive green), it had to be kept constant for reproducible results. The same had to be applied for the capturing parameters and subsequent electronic treatments of the image. When individually chosen for each image (e.g., white adjust and spot amplification tools), differences were evident (Fig. 7).

The immersion of cut plates was associated with special effort when using an automated immersion device due to the insufficient immersion of the reduced plate height into the liquid level. Piezoelectric spraying was newly applied and found highly advantageous for reagent application on cut plates. Both plate parts were simply put together and piezoelectrically sprayed with the anisaldehyde sulfuric acid reagent.

Workflow I (Fig. 7) is recommended for the 2-step separation because it is the most efficient procedure with regard to analysis time (20 min) and solvent consumption (10 mL). The impurity study is processed. However, there are further options to achieve a satisfying band shape under such circumstances. For workflow II (Fig. 7), the development was first performed with *n*-hexane-ethyl acetate-glacial acetic acid (7:3:1) up to 85 mm for separation of steviol (*hR*_F_ 80) and isosteviol (*hR*_F_ 87), and secondly with acetonitrile-water (4:1) up to 60 mm for separation of steviol glycosides. The separation required only 14 mL mobile phase solvents, but took almost 1 h. For workflow III, the development was faster (20 min) but consumed a higher solvent volume (95 mL). The sample solutions were applied at a higher position on the HPTLC plate and developed with a higher volume of the apolar mobile phase for separation of steviol and isosteviol (ESM Fig. S12). Then, the chromatogram was cut at 70 mm and the lower larger plate part was turned by 180°. Thus, the previous application line still containing the steviol glycosides (now positioned at 10 mm) was developed with the polar mobile phase for separation of the steviol glycosides. Due to the high flexibility of the HPTLC technique, there are always more options to circumvent or solve a problem.

## Conclusions and outlook

A HI-HPTLC-UV/Vis/FLD method was developed and exemplarily applied to *Stevia* leaf extracts and 20 different food products. As shown, it was not only robust with regard to the different matrices but also powerful enough to provide further information in questionable cases. In a straightforward manner, the method was optionally combinable with HRMS or other derivatization reagents or bioassays. This offered a high degree of flexibility to solve the respective analytical issue. It was demonstrated to be suited as a fast screening, but can also be combined with HRMS for confirmation or quantification. The multi-imaging provided different chemical, biochemical, and biological profiles that delivered comprehensive information on bioactive components in *Stevia* leaf extracts. In contrast to the 19 different bioactive compounds found in the more natural leaf extracts, most activities were not existent for the steviol glycosides. This revealed that *Stevia* leaves may provide health benefits via subtle impact on several metabolic pathways and thus homeostasis, in contrast to the highly sweet, but otherwise poorly active steviol glycosides.

This bioanalytical tool closes a major gap in the current analytical toolbox. Since a picture is worth a thousand words, it can better illustrate and evaluate the pros and cons that originate from multipotent multicomponent botanical mixtures. Exploiting a minimalistic sample preparation, multi-imaging and non-targeted bioprofiling, any product change, falsification, or adulteration is figured out faster compared with most less flexible status quo methods. Are we analytically on the right track? Do the mainstream techniques allow for a cost-efficient bioanalytical screening of complex food? Do we dare to ask the right questions? Do we need information on bioactive unknown unknowns (major portion of features/compounds is unidentified)? Can we discover unknown trace compounds of high activity potential with the given status quo methods? In particular, the bioprofiling substantially contributes to solve the daunting challenge of food safety along the global product chain. It directly points to active compounds that matter, which should be of interest for food control and safety, but are currently out of the focus.

## Electronic supplementary material

ESM 1 (PDF 3.19 mb)
