# Supplementary material for: HI-HPTLC-UV/Vis/FLD-HESI-HRMS and bioprofiling of steviol glycosides, steviol, and isosteviol in Stevia leaves and foods
Source: Anal Bioanal Chem. 2020 Apr 23;412(24):6431–48. doi: 10.1007/s00216-020-02618-4 (PMC7442773; doi:10.1007/s00216-020-02618-4)
Supplement: Supplementary file 1 — (PDF 3.19 mb) [file 216_2020_2618_MOESM1_ESM.pdf]

Analytical and Bioanalytical Chemistry

Electronic Supplementary Material

HI-HPTLC-UV/Vis/FLD-HESI-HRMS and bioprofiling of steviol glycosides, steviol, and isosteviol in *Stevia* leaves and food

G.E. Morlock[a,*], J. Heil[a]

[a]Institute of Nutritional Science, Chair of Food Science, and TransMIT Center for Effect-Directed Analysis, Justus Liebig University Giessen, Heinrich-Buff-Ring 26-32, 35392 Giessen, Germany

*Corresponding author. Tel.: +49 641 9939141; fax: +49 641 9939149.

E-mail address: Gertrud.Morlock@uni-giessen.de (G.E. Morlock).

https://orcid.org/0000-0001-9406-0351

Table of content (shortened legends)

**Table S1**	Compilation of *Stevia* leave products I to IV and 20 different food products	Page S-3
**Table S2**	Compilation of structural information on isosteviol, steviol and the twelve steviol glycosides	Page S-5
**Fig. S1**	Study of the addition of 2-aminoethyl diphenylborinate	Page S-6
**Fig. S2**	Study of the most sensitive detection of steviol glycosides	Page S-7
**Fig. S3**	Example of measuring the HPTLC-HESI$^{-/+}$-HRMS spectra of the 12 steviol glycosides	Page S-8
**Fig. S4**	Chemical profiles of *Stevia* leaf products I-IV	Page S-9
**Fig. S5**	Study of the colors of the individual steviol glycosides after derivatization with the anisaldehyde sulfuric acid reagent	Page S-10
**Fig. S6**	Performance qualification of the *in situ* assays showing the positive control	Page S-11
**Fig. S7**	Proof of any side reaction of the chromogenic substrate other than with the enzyme product	Page S-12
**Fig. S8**	Confirmation of the β-glucuronidase and AChE inhibiting effect of compounds 11 and 19 below the solvent front in the ethanol-water extract III	Page S-13
**Fig. S9**	HPTLC chromatograms of the reagent sequence, *i.e.* three different reagents in sequence on the same plate, for proof of the falsified sample E.N.D. Stevia (ID 6)	Page S-14
**Fig. S10**	Analysis of steviol glycosides, 19 saccharides and 5 different derivatives detected by (a) derivatization with the diphenyl amine aniline phosphoric acid reagent and (b) *A. f.* bioassay	Page S-15
**Fig. S11**	Separation of steviol (S) and isosteviol (IS)	Page S-16
**Fig. S12**	Workflow III with first separation of steviol and isosteviol taking 6 min	Page S-17

Table S1 Compilation of *Stevia* leave products I to IV and 20 different food products, including parameters of sample preparation and application volumes for detection via the 2-naphthol sulfuric acid reagent (for an 8-mm band, application volumes were multiplied by 1.5 or 2, for detection via anisaldehyde sulfuric acid reagent by 4 and for detection via primuline reagent by 7)

Stevia	**ID**	**Product of JLU Giessen, Germany, or confidential supplier**	**Amount (mg)**	**Volume (mL) ethanol-water 4: 1 or ethyl acetate**	**µ-Diluted (in vial) in methanol**	**Applied (µL/ 6-mm band)**
Dried leaf (powder)	**I**	*Stevia rebaudiana* fol. conc. S2934	10	1	-	1.5 or 3
	**II**	*Stevia* leaf organic S8995				
	**III**	*Stevia* leaf conventional S8996				
	**IV**	Koyro No. 4				
**Food type**		**Product name; Manufacturer**		**methanol - water 9:1**		
Powder	**1**	Stevie; Industrias Majota, La Paz, Bolivia	20	10	-	1
	**2**	Steviolglykoside, Granulat 90%; Raab Vitalfood, Rohrbach, Germany				
	**3**	Steevia-Steviosid-Extrakt, Gesund & Leben, Stockach, Germany			1:10	
	**4**	SweetLeaf, Organic Stevia Sweetener; Wisdom Natural Brands, Gilbert, AZ, USA			-	
	**5**	*Stevia;* La Bolivianita, San Miguel, Bolivia				
	**6**	E.N.D. *Stevia*; Los Yungas, La Paz, Bolivia				
Crystals	**7**	Nevella, Low Calorie Sweetener, *Stevia,* sticks*;* Heartland Food Products Group, Carmel, IN, USA	20	5	-	3
	**8**	Dolciando Dolcificante con derivati della Stevia; Eurospin Italia, S. Marino, Italy				
	**9**	Green sugar, dulce natural, zero calorii; Sc Laboratoarele Remedia, Bucuresti, Romania				
	**10**	ERYLITE Stevia 200; Jungbunzlauer, Ladenburg, Germany				
	**11**	ERYLITE Stevia 400; Jungbunzlauer, Ladenburg, Germany				1.5

Tablet (ca. 60 mg/tab)	**12**	*Stevia,* 300 Tabs; Raab Vitalfood, Rohrbach, Germany	50	10	1:10	1
	**13**	Steevia TAB´s, 300 Tabs; Gesund & Leben, Stockach, Germany				
	**14**	*Stevia,* 100 Tabs; dm drugstore chain, Giessen, Germany				
Tea	**15**	Sweet Gingerdream; Oasis Teehandel, Bondorf, Germany	500	20	-	1
	**16**	Sweet Evening; Oasis Teehandel Bondorf, Germany				
Crystals	**17**	*Stevia;* www.mgo-life.com	20	5	-	3
Tablet	**18**	Canderel *Stevia*-Green Tab; Valora, Neuendorf, Switzerland	40	5	1:5	1
Cereal porridge	**19**	Tee Fee Wunderflocken, Bio Kindermüsli; la marchante, Frankfurt, Germany	250	1	-	1
Liquid extract	**20**	Steevia Dulce; Gesund & Leben, Stockach, Germany	100 µL aqueous	10 methanol	-	1

Table S2 Compilation of structural information on isosteviol, steviol and twelve steviol glycosides, ordered according to decreasing *hR*F value almost analogous to increasing saccharide moieties (No. 0-6)

Steviol

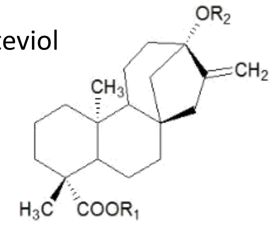

Isosteviol

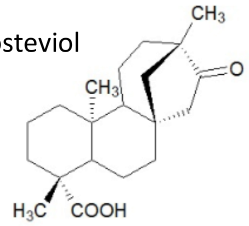

No.	Substance	*hR*F	Theoretical *m/z* [M-H]⁻ (*vs.* measured in Fig. 3)	Mass error (ppm)	R1	R2
0	Isosteviol	95	317.2122		see structure	
	Steviol	95	317.2122		-H	-H
2	Rubusoside	83	641.3179	-1.13	-β-Glc	-β-Glc
	Steviolbioside	78	641.3179	-1.29	-H	-β-Glc-β-Glc (2→1)
3	Rebaudioside B	72	803.3707	-1.22	-H	-β-Glc-β-Glc (2→1)<br>I<br>β-Glc (3→1)
	Dulcoside A	60	787.3758	-1.25	-β-Glc	-β-Glc-α-Rha (2→1)
	Stevioside	48	803.3707	-1.51	-β-Glc	-β-Glc-β-Glc (2→1)
4	Rebaudioside C	44	949.4286	-0.63	-β-Glc	-β-Glc-α-Rha (2→1)<br>I<br>β-Glc (3→1)
	Rebaudioside A	32	965.4235	-0.76	-β-Glc	-β-Glc-β-Glc (2→1)<br>I<br>β-Glc (3→1)
5	Rebaudioside I	24	1127.476	-0.09	-β-Glc-ß-Glc (3→1)	-β-Glc-β-Glc (2→1)<br>I<br>β-Glc (3→1)
4	Rebaudioside E	22	965.4235	-0.73	-β-Glc-β-Glc (2→1)	-β-Glc-β-Glc (2→1)
6	Rebaudioside M	16	1289.529	-0.07	-β-Glc-β-Glc (2→1)<br>I<br>β-Glc (3→1)	- β-Glc-β-Glc (2→1)<br>I<br>β-Glc (3→1)
5	Rebaudioside D	15	1127.476	-0.57	-β-Glc-β-Glc (2→1)	-β-Glc-β-Glc (2→1)<br>I<br>β-Glc (3→1)
6	Rebaudioside N	6	1273.534	-0.05	-β-Glc-α-Rha (2→1)<br>I<br>β-Glc (3→1)	-β-Glc-β-Glc (2→1)<br>I<br>β-Glc (3→1)

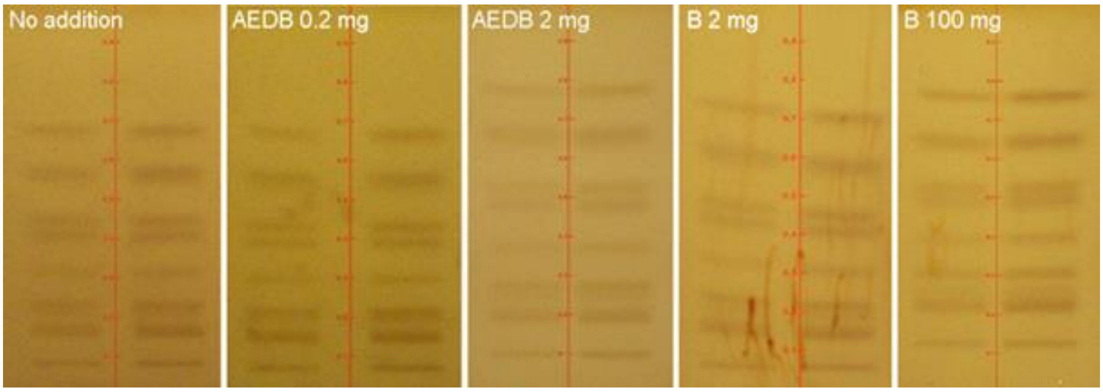

Fig. S1 Study of the addition of 2-aminoethyl diphenylborinate (AEDB, 0.2 and 2 mg) and boric acid (B, 2 and 100 mg) on the HILIC separation of steviol glycosides (60 and 100 ng/band) with acetonitrile – water 5:1, detected after derivatization with 2-naphthol sulfuric acid reagent and documented at white light illumination

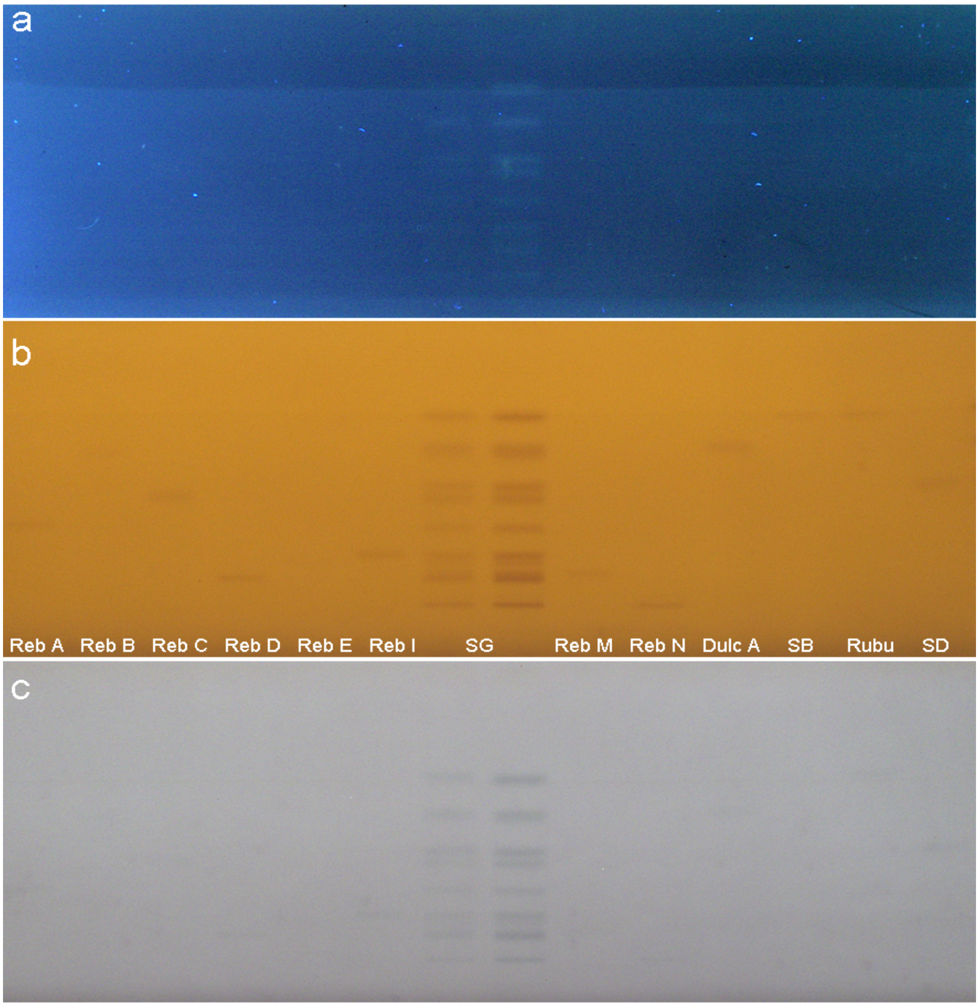

Fig. S2 Study of the most sensitive detection of steviol glycosides (SG mixture 100 and 200 ng/band each; individually applied ones 30 ng/band) after HILIC separation with acetonitrile – water 5:1, using three different derivatization reagents: (a) *p*-aminobenzoic acid reagent (0.5% 2-aminoethyl diphenylborinate in methanol, followed by heating at 200°C for 5 min) documented at UV 366 nm, (b) 2-naphthol sulfuric acid reagent and (c) diphenyl amine aniline phosphoric acid reagent (2% each in methanol - *o*-phosphoric acid 9:1), both latter documented at white light illumination

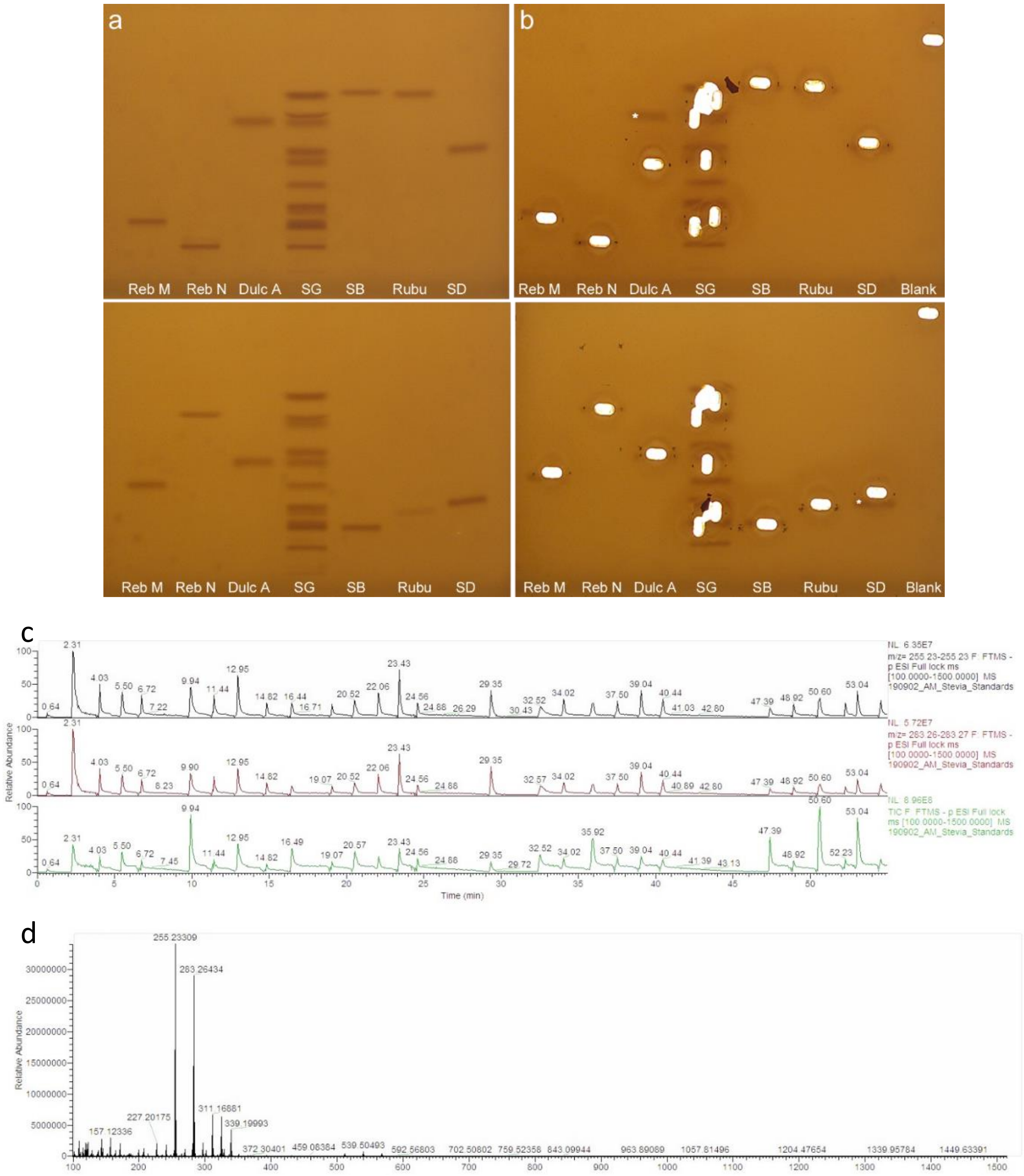

Fig. S3 Example of measuring the HPTLC-HESI$^{-/+}$-HRMS spectra of the 12 steviol glycosides (250 ng/elution) after HILIC separation with acetonitrile – water 5:1: (a) one plate part was derivatized with 2-naphthol reagent and used for marking the zones on the second plate half; (b) after elution, this plate part was also derivatized with 2-naphthol reagent to check the proper position of the elution head (two zones marked* were analyzed again due to wrong positioning); on the track of the steviol glycoside mixture (SG), the plate was turned by 90° to simulate coelution (two bands at one go) and documented at white light illumination; (c) extracted ion current (main signals at *m/z* 255.23 and 283.26) and total ion current chronograms of (d) background spectrum

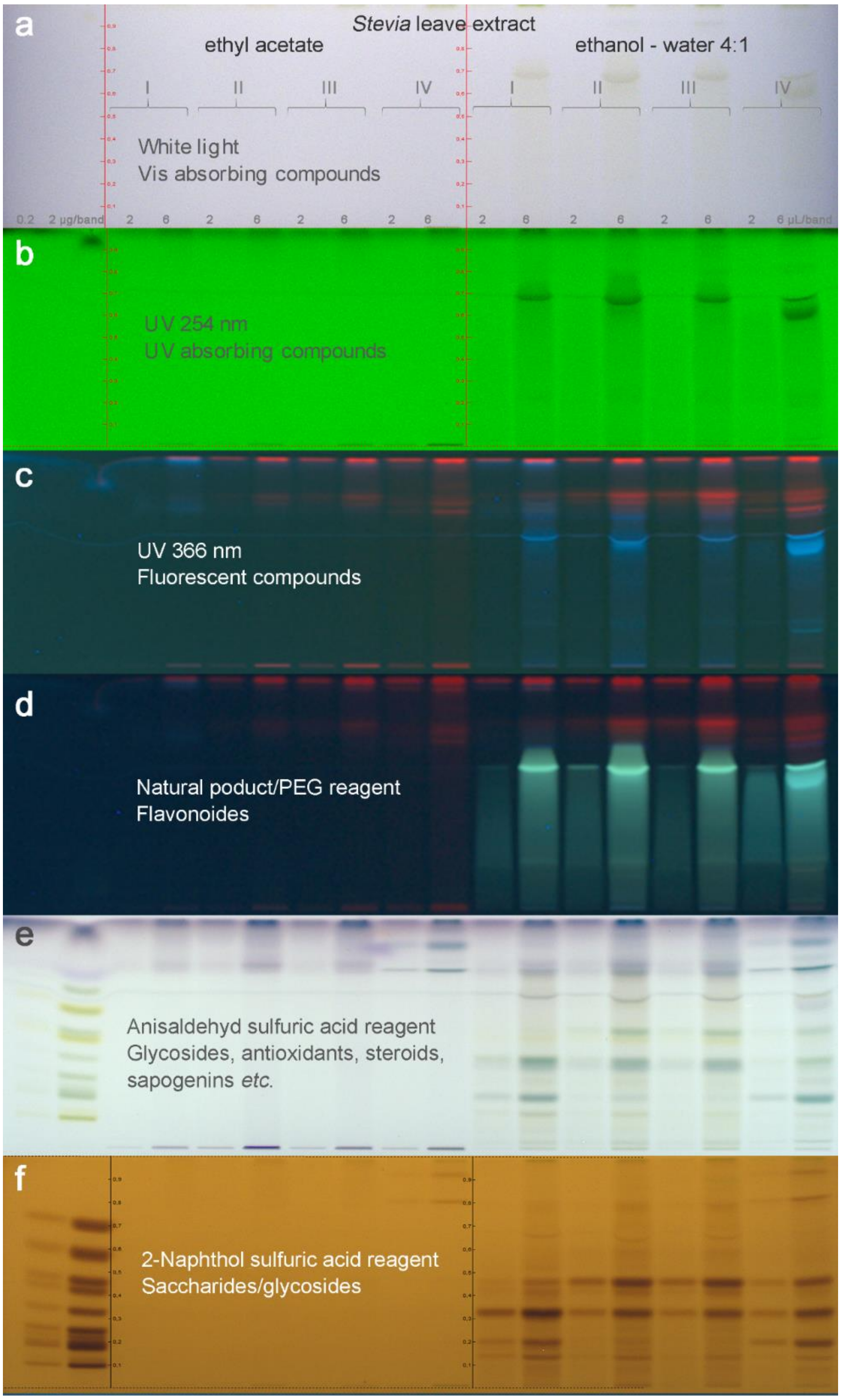

Fig. S4 Chemical profiles of *Stevia* leaf products I-IV (extracted with either ethyl acetate or ethanol – water 4:1; 10 mg/mL each; 2 and 6 µL/8-mm band each): HPTLC chromatograms (on HPTLC plate silica gel 60 F_{254} with acetonitrile – water 5:1) at (a) white light illumination, (b) UV 254 nm, (c) UV 366 nm as well as after derivatization with (d) natural product reagent (0.5% 2-aminoethyl diphenylborinate in methanol, followed by 5% methanolic PEG 400 solution, dried in a stream of warm air) at UV 366 nm (green diffuse tailing may be caused by phenolic acids that are not focused with this neutral mobile phase), (e) anisaldehyde sulfuric acid reagent and (f) 2-naphthol sulfuric acid reagent, both latter at white light illumination after heating (110 °C, 5 min)

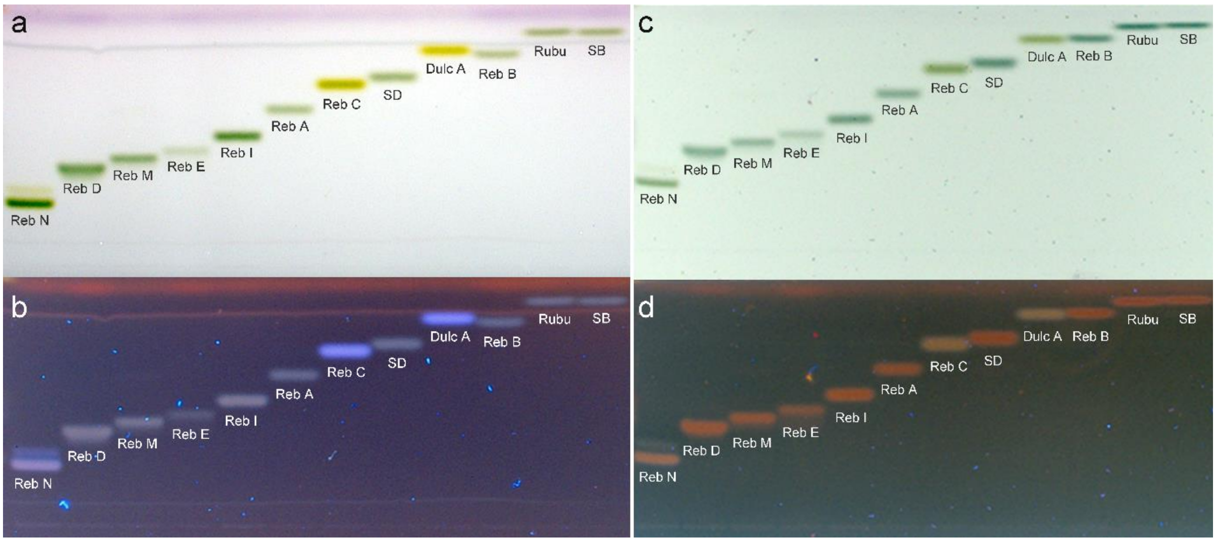

Fig. S5 Study of the colors of the individual steviol glycosides (2 µg/band each) after derivatization with the anisaldehyde sulfuric acid reagent (heated 110 °C, 5 min) showing (a) yellow (the three rhamnose-containing glycosides) and green colors at white light illumination and (b) the yellow bands with a more intense blue fluorescence at UV 366 nm, helpful to distinguish especially critical pairs, when separated on HPTLC plate silica gel 60 F_{254} with acetonitrile – water 4:1 in twin trough chamber up to 60 mm; (c and d) the same but on prewashed plate without fluorescence indicator; prewashing had a negative impact on zone resolution of critical pairs and color expression, but positive impact on plate background (cleanness); the 82%-purity of Reb N is evident by the second band above especially at UV 366 nm; differences in the signal/band intensities are more pronounced when compared to the 2-naphthol sulfuric acid reagent (Fig. S4f)

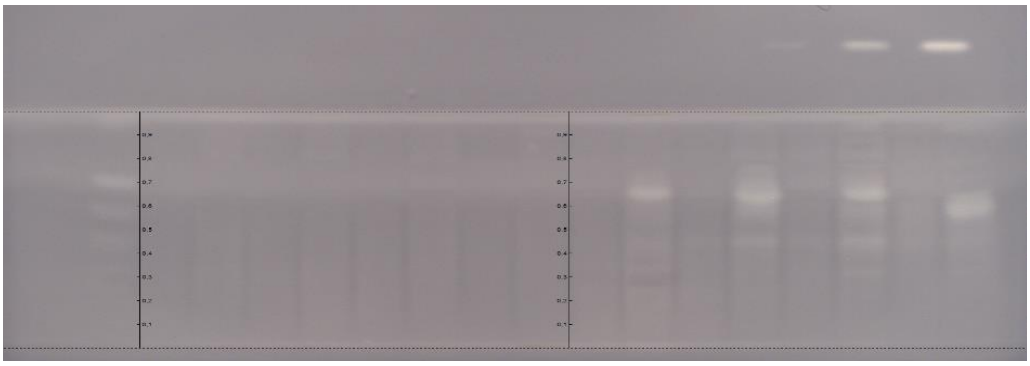

Fig. S6 Performance qualification of the *in situ* assays showing the positive control as described in the materials part applied as 8-mm bands above the solvent front on the upper right plate part to document the proper assay performance, exemplarily shown for the tyrosinase inhibition assay with 0.1-mg/mL ethanolic kojic acid applied at 1, 3 and 6 µL/band

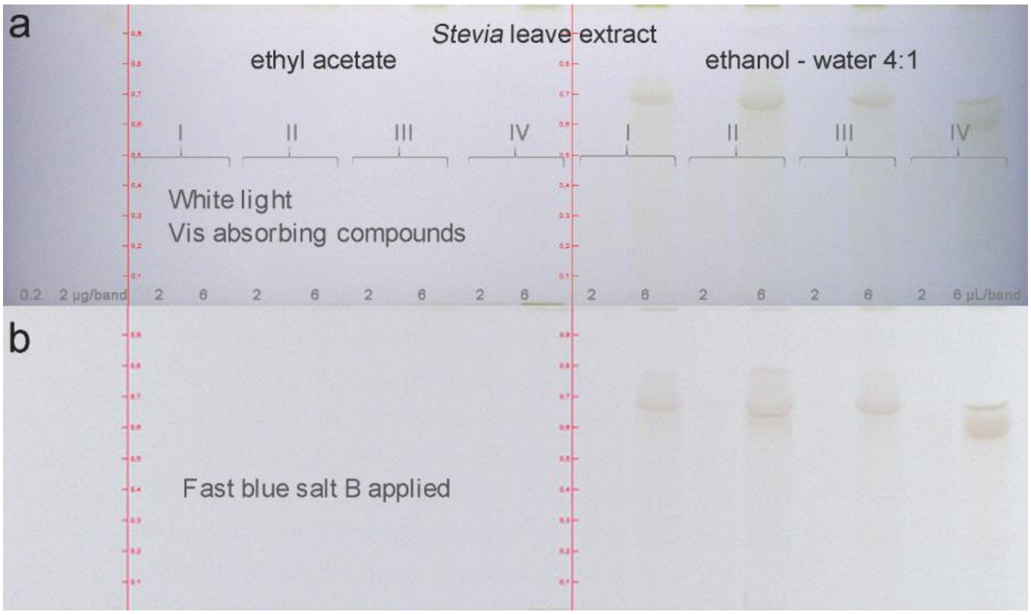

Fig. S7 Proof of any side reaction of the chromogenic substrate other than with the enzyme product: immersion of the chromatogram in the Fast Blue B salt substrate solution (used in the α-glucosidase, β-glucosidase, AChE and BChE enzyme assays): (a) HPTLC chromatogram of *Stevia* leaf products I-IV (extracted with either ethyl acetate or ethanol – water 4:1; 10 mg/mL each; 2 and 6 µL/8-mm band each) on HPTLC plate silica gel 60 with acetonitrile – water 5:1 in twin trough chamber up to 60 mm, documented at white light illumination *versus* (b) after derivatization with Fast Blue B salt reagent (1% Fast Blue B salt in water - methanol – dichloromethane, 2:5:3) showing no difference

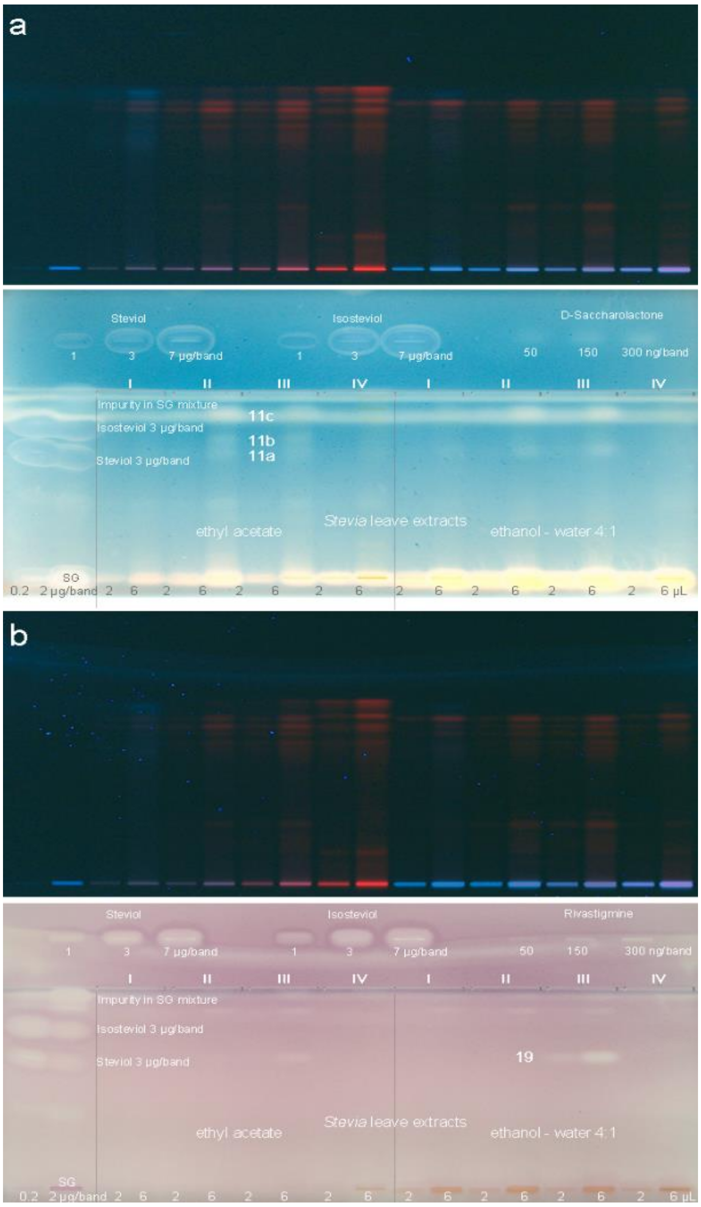

Fig. S8 Confirmation of the (**a**) β-glucuronidase inhibiting effect of compound zone **11** and (**b**) AChE inhibiting effect of compound zone **19**, both below the solvent front in the ethanol-water extract III: HPTLC images at UV 366 nm, and after the respective assay, at white light illumination; same scheme as in Fig. 4h, but developed with a mobile phase of reduced elution power, *i.e.* *n*-hexane – ethyl acetate – glacial acetic acid 7:4:0.2 (steviol glycosides remained at the start zone) and additional overspraying (3 µg/band each on both SG bands of 0.2 and 2 µg/band) and application of steviol and isosteviol as band pattern of 1 - 7 µg/band above the solvent front, along with the respective positive control (D-saccharolactone or rivastigmine); any formic acid traces that remained on the chromatogram after the development were neutralized by piezoelectric spraying with 2 mL phosphate buffer (8 g disodium hydrogen phosphate in 60 mL water with addition of citric acid 0,M to pH 7.5, ad 100 mL; yellow nozzle, level 6, Derivatizer, CAMAG)

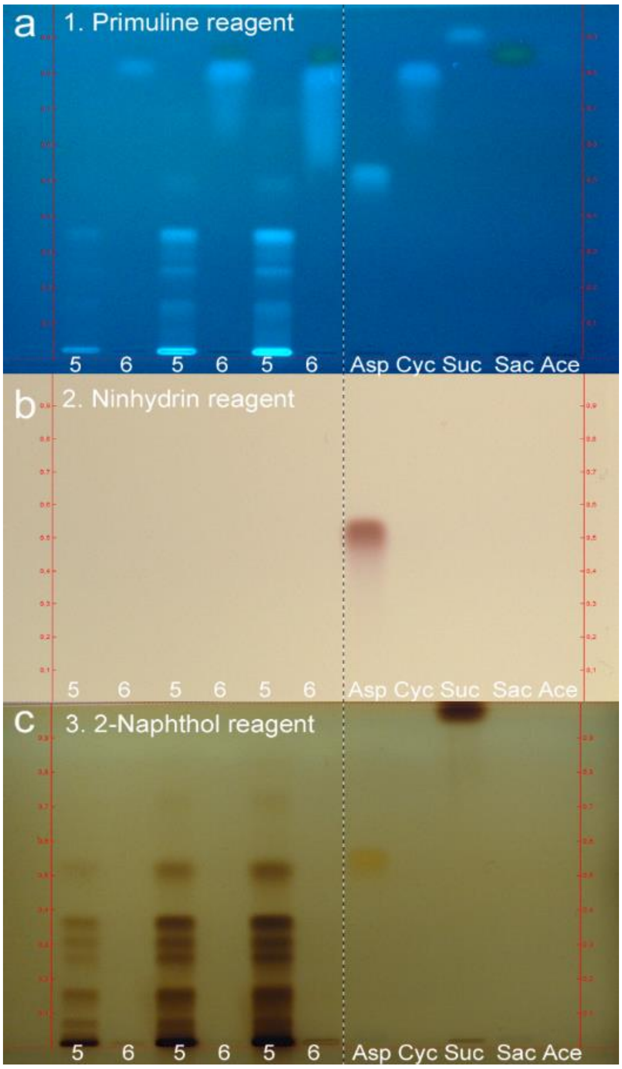

Fig. S9 HPTLC chromatograms of the reagent sequence, *i.e.* three different reagents in sequence on the same plate, for proof of the falsified sample E.N.D. Stevia (ID 6, Table S1) to contain sodium cyclamate and saccharine instead of any steviol glycoside: Powder samples ID 5 and ID 6 (1, 5 and 10 µL/band each) along with sweeteners (aspartame, Asp; sodium cyclamate, Cyc; sucralose, Suc; saccharine, Sac and acesulfam potassium, Ace; 4 µg/band each) were detected by successive derivatization of the same plate with (a) primuline reagent at UV 366 nm, (b) ninhydrin reagent and (c) 2-naphthol reagent; both latter at white light illumination; HPTLC separation (G. Morlock, G. Sabir, private communication)

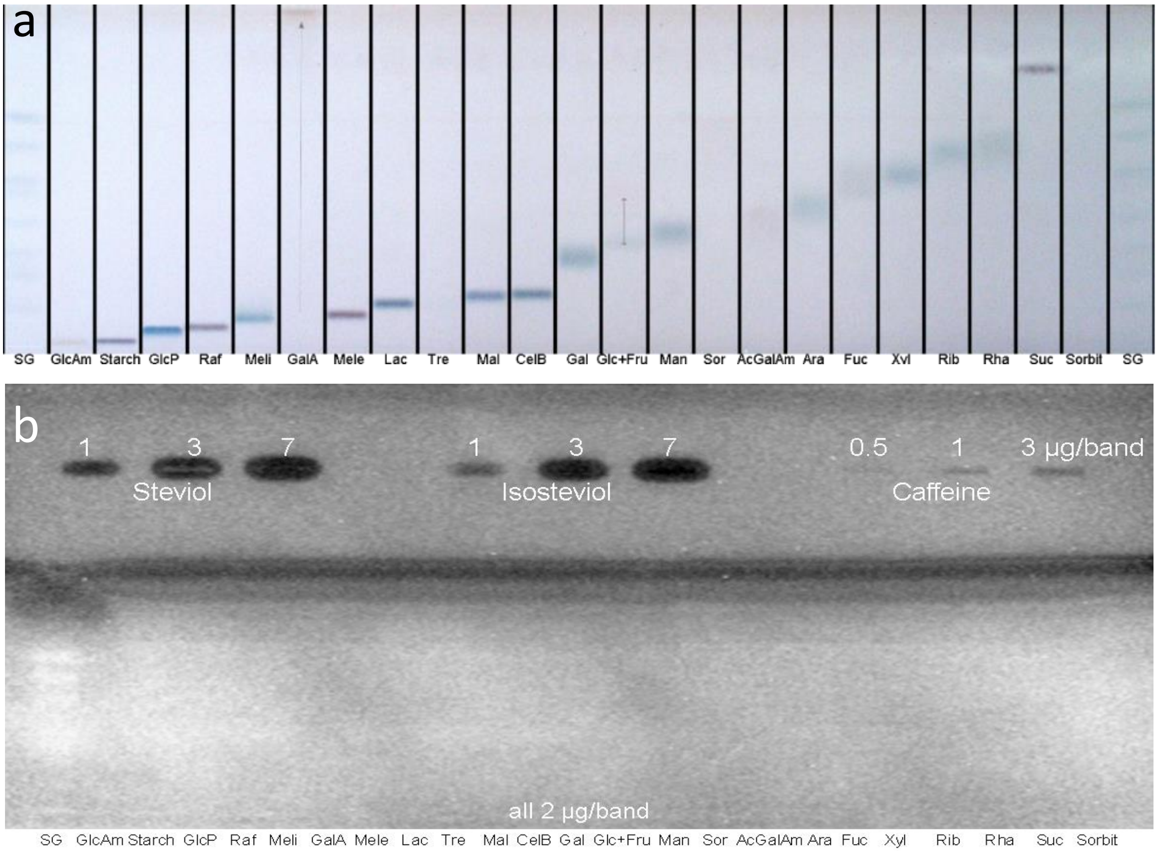

Fig. S10 Analysis of steviol glycosides (SG, 150 ng/band each), 19 saccharides and 5 different derivatives (for a, 200 ng/band versus for b, 2 µg/band each), such as gylcosyl amines (Am; acetylated, ac), gylcosyl phosphates (P), glycuronic acids (A) and sugar alcohols (not detectable) on HPTLC plate silica gel 60 with acetonitrile – water 5:1, all ordered according to ascending hR_F value (for a, plus 0.2 mg 2-aminoethyl diphenylborinate in mobile phase that separates Fru from Glc and elutes GalA to front) in twin trough chamber up to 60 mm detected after (a) derivatization with the diphenyl amine aniline phosphoric acid reagent (2% each in methanol – *o*-phosphoric acid 9:1) and documented at white light illumination as well as (b) *A. f.* bioassay (bioluminescence as greyscale image): the 19 saccharides and 5 different derivatives (2 µg/band each) showed no bioactive response in contrast to steviol and isosteviol (1 - 7 µg/band each) applied along with the positive control caffeine (0.5 - 3 µg/band) in the upper plate part

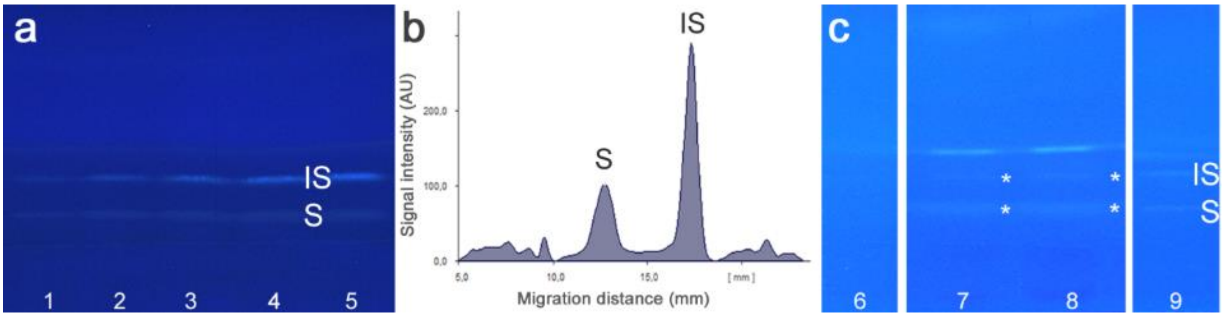

Fig. S11 Separation of steviol (S) and isosteviol (IS): Plate cut in the middle, followed by development of the upper plate piece (S/IS located at ca. 8 mm as new start line, as near solvent front at 60 mm before plate cut) with 5 mL *n*-hexane – glacial acetic acid 19:1, derivatization with the primuline reagent and documentation at UV 366 nm; (a) separation of S/IS (tracks 1-5, 150-4500 ng/band), (b) densitogram at 366/>400 nm, and (c) blank (track 6) and proof of presence of S/IS (marked*) in sea buckthorn candy (tracks 7 and 8)

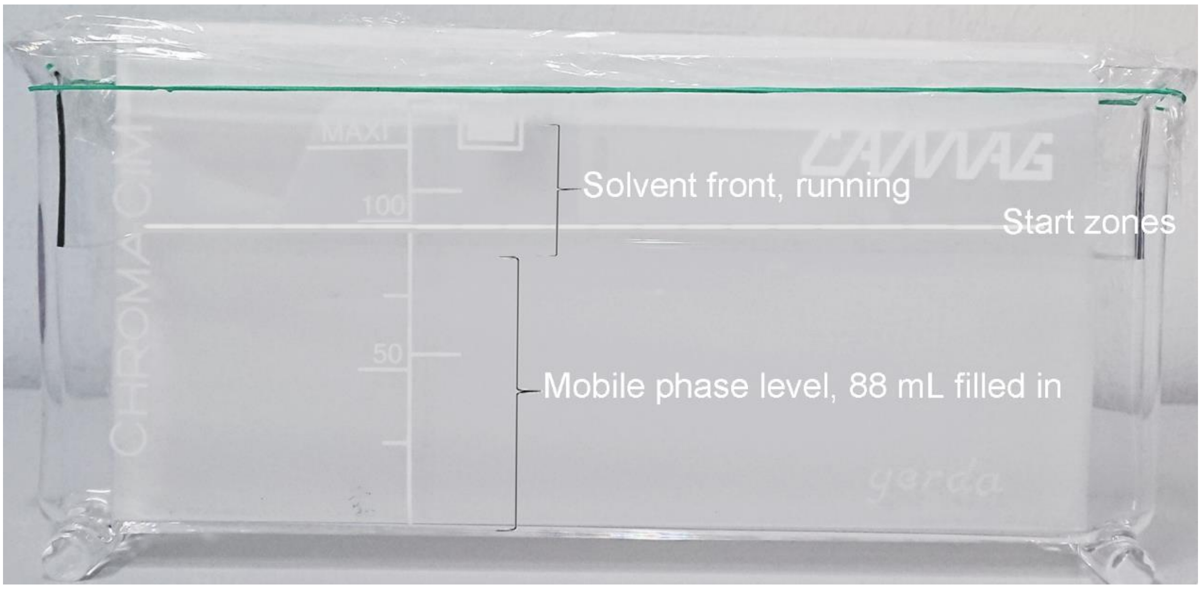

Fig. S12 Workflow III with first separation of steviol and isosteviol taking 6 min: The sample solutions were applied at 60 mm on the HPTLC plate (start zones at a higher position as usual) and developed with 88 mL *n*-hexane – ethyl acetate – glacial acetic acid, 7:3:1 (*V*/*V*) up to the plate edge in the Chromacim Immersion Chamber as depicted (covered by an airtight foil to avoid evaporation, because the HPTLC plate is too high for the normal glass lid); the start zones should be at least 5 mm above the liquid level to avoid their washing out into the liquid
